# Sampling Terrestrial Environments for Bacterial Polyketides

**DOI:** 10.3390/molecules22050707

**Published:** 2017-04-29

**Authors:** Patrick Hill, Graham W. Heberlig, Christopher N. Boddy

**Affiliations:** 1 Department of Biology, University of Ottawa, Ottawa, ON K1N 6N5, Canada; phill@uottawa.ca; 2 Department of Chemistry and Biomolecular Sciences, University of Ottawa, Ottawa, ON K1N 6N5, Canada; ghebe104@uottawa.ca

**Keywords:** polyketides, bioprospecting, microbial ecology

## Abstract

Bacterial polyketides are highly biologically active molecules that are frequently used as drugs, particularly as antibiotics and anticancer agents, thus the discovery of new polyketides is of major interest. Since the 1980s discovery of polyketides has slowed dramatically due in large part to the repeated rediscovery of known compounds. While recent scientific and technical advances have improved our ability to discover new polyketides, one key area has been under addressed, namely the distribution of polyketide-producing bacteria in the environment. Identifying environments where producing bacteria are abundant and diverse should improve our ability to discover (bioprospect) new polyketides. This review summarizes for the bioprospector the state-of-the-field in terrestrial microbial ecology. It provides insight into the scientific and technical challenges limiting the application of microbial ecology discoveries for bioprospecting and summarizes key developments in the field that will enable more effective bioprospecting. The major recent efforts by researchers to sample new environments for polyketide discovery is also reviewed and key emerging environments such as insect associated bacteria, desert soils, disease suppressive soils, and caves are highlighted. Finally strategies for taking and characterizing terrestrial samples to help maximize discovery efforts are proposed and the inclusion of non-actinomycetal bacteria in any terrestrial discovery strategy is recommended.

## 1. Introduction

Polyketide natural products are exquisite molecules, often with extraordinary and diverse structures ranging from macrolides to polyethers and polyphenols. They typically bind with high affinity and selectivity to a biological target (usually a protein or RNA) and can cross biologically relevant membranes, enabling them to modulate the biology of organisms. These features have made polyketide natural products an indispensable resource for the discovery and development of new pharmacological agents including the antibiotics, like erythromycin A and tetracycline, antitumor agents, like doxorubicin; and the anti-fungal drugs, like amphotericin B [1].

While structurally diverse, all polyketides are related by their highly conserved biosynthetic origins. The polyketide backbone is assembled, analogously to fatty acid biosynthesis, through sequential additions of two carbon building blocks (ketide units) derived from malonyl-CoA. This assembly is catalyzed by enzymes called polyketide synthases (PKS). In bacteria these polyketide synthases typically occur with one of two different architectures, modular type I polyketide synthases and the iterative type II polyketide synthases.

The type I PKS pathways contain multiple modules consisting of ketosynthase (KS) domains, which catalyze the addition of ketide units to the growing polyketide chain via decarboxylative Claisen condensation of an acyl carrier protein (ACP)-linked malonyl [2,3]. In addition to the KS and ACP many type I PKS pathways also possess an acyltransferase (AT) domain embedded into each module which loads the appropriate malonyl extender unit onto the ACP. The trans AT type I PKS pathways possess a separate standalone AT domain responsible for loading the ACP domains [4]. In addition to these catalytic domains which extend the polyketide chain, type I PKS pathways can also possess reductive domains such as a ketoreductase (KR), dehydratase (DH), and enoylreductase (ER) domains that reduce the growing polyketide intermediate, similar to mammalian fatty acid biosynthesis prior to the next round of polyketide elongation [2,3]. Elongation continues until eventual the completed polyketides is release from the ACP by a thioesterase (TE) domain [5]. Type I PKS are responsible for the production of a wide variety of bioactive compounds including the antibiotic erythromycin and the antifungal amphotericin B.

Type II polyketide synthases are most frequently found in the order of bacteria actinomycetales and function similarly to bacterial fatty acid synthases. Like type I PKS, these synthases rely on a KS domain, which heterodimerizes with a protein called chain length factor (CLF), and an ACP domain. A malonyl acyltransferase (MAT) domain is used to load malonyl onto the ACP for decarboxylative condensation with the growing KS-bound polyketide chain. Unlike type I PKS, these pathways typically use these four catalytic domains iteratively and possess a single reductive domain [3,6]. Typical products from type II PKS pathways are aromatic compounds that are often highly tailored to generate complex molecules such as the antibiotic tetracycline or the anticancer agent doxorubicin.

Often type I polyketide synthases form hybrid pathways with non-ribosomal peptide synthetases, producing natural products containing both polyketide portions and peptide portions [7,8,9]. The anticancer agent epothilone B is a prime example of a product from this type of pathway. In this review article we broadly define bacterial polyketides as compounds produced by type I and type II polyketide synthase biosynthetic pathways as well as hybrid polyketide-non-ribosomal peptide pathways.

The discovery of new polyketides has slowed noticeably since the late 1980s as the traditional method of discovery, isolating and cultivating bacteria (usually Actinomycetes, and especially their genus *Streptomycetes* [1]) from soil, found fewer and fewer new polyketides. At the same time bacterial resistance to antibiotics has been increasing. To restart polyketide discovery, bioprospectors are using methods that improve or replace bacterial cultivation and sampling alternative environments to soil. Both of these approaches mean that it is important to know how bacterial polyketides are distributed to sample them efficiently.

Soil cultivation methods can be scaled up to screen millions of isolates a year to find rare polyketides [10]. Sequencing of the genomes of isolated bacteria shows that most polyketide genes are “cryptic”, i.e., not expressed under normal cultivation conditions. In some cases these cryptic pathways have been coaxed to express by changing the cultivation conditions [11], adding antibiotics that stimulate polyketide production [12], overexpressing regulatory genes [13], or blocking competing pathways [14]. Alternatively these cryptic pathways can be expressed in heterologous hosts, enabling new compound isolation. While this approach has a number of technical hurdles, including ensuring transcription, codon usage, protein folding, ensuring the presence of precursors, and concerns that the polyketide product itself may kill the host [15], there have been some major discoveries in this area [16].

While most bacteria in soil cannot be readily grown, the cultivatable fraction of the soil bacterial community can be increased by keeping bacteria in contact with the soil in buried “chips” for weeks [17]. To circumvent cultivation, bacterial DNA can be extracted from soil and heterologously expressed in bacteria that can grow in the laboratory [18,19].

These new techniques are changing the way polyketides are discovered (for reviews see [20,21,22,23,24,25]), but they require as much or more time and effort for each sample as the lower tech isolation methods that they replace, so sampling efficiently is important. The problem of how to sample is no longer limited to soil. Bioprospectors can and should consider the many alternatives to soil such as caves, insects and desert soils. Bioprospectors need to compare the microbial polyketide producers within and between environments to find the most polyketide diversity with the fewest samples.

**Figure 1:**
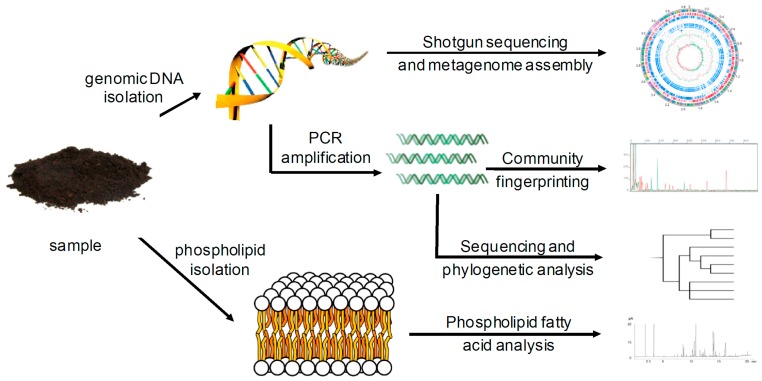
Microbial ecology approaches used to examine both cultivatable and uncultivatable fraction of the microbial community structure. This includes molecular methods like shotgun metagenome sequencing, community fingerprinting of polymerase chain reaction (PCR) products, sequencing of PCR products, and phospholipid fatty acid analysis.

Until recently it was impossible to compare the bacterial community structure between environments. It had long been known that only one of every hundred to thousand bacteria from the environment that could be seen through a microscope could be grown in the laboratory [26]. In 1990 DNA was extracted from soil, denatured, and its reassociation time measured, allowing researchers to estimate that there were the equivalent of 4000 *E. coli* genomes in each gram of the soil [27]. This was one of the first uses of cultivation-independent, nucleic acid-based methods to study soil bacteria. Modern nucleic acid based methods usually use the polymerase chain reaction (PCR) to measure which bacteria are in the soil and estimate how diverse the bacterial community is (Figure 1).

PCR products amplified from soil DNA templates typically contain thousands or more different sequences. Cloning and sequencing with Sanger sequencing (e.g., [28]) was used until the early 2000s to sequence these amplicons. Due to the intrinsically low throughput of Sanger sequencing and thousands to hundreds of thousands of bacterial species in soil, it was difficult to get a full picture of the bacterial community of a single sample, let alone to compare samples. An alternative was to separate the PCR amplicons by gel electrophoresis to get a “community fingerprint” and compare fingerprint patterns to get a rough idea of how bacterial communities differed. Gel fingerprinting can separate amplicons several ways, including gradients of temperature or urea, which separate amplified DNA by guanine/cytosine (GC) content (e.g., Denaturing Gradient Gel Electrophoresis, DGGE [29]), or cutting amplicons into smaller fragments by restriction endonucleases and separating by size (e.g., Terminal Restriction Fragment Length Polymorphism, T-RFLP [30]). Community fingerprinting gives less information than Sanger sequencing, but can quickly and cheaply compare many samples (Figure 1).

In the 2000s next generation sequencing replaced Sanger sequencing and fingerprinting of PCR products. With next generation sequencing thousands to hundreds of thousands of different amplicons could be sequenced from a single sample and many samples could be multiplexed together to give enormous data sets [31]. This increased the volume of sequencing results and the number of sequenced genomes. Researchers could even sequence unamplified DNA from the soil metagenome (Figure 1) and estimate which phylogenetic groups it belonged to [32].

Nucleic acids are not the only biomolecules that can be used to measure microbial community structure. Analysis of phospholipid fatty acids (PLFA) from the cell membranes of soil microbial communities began in the 1980s (Figure 1) [33]. Like nucleic acid methods, it provides information on the uncultivated majority of bacteria. While PLFA has largely been replaced by next generation sequencing, there is a lot of PLFA data in the literature. Unlike nucleic acid sequencing, PLFA cannot identify bacteria at the genus or species level and is often specific to groups that are even broader than phyla (e.g., Gram-positive bacteria). In spite of this there are advantages to PLFA. It is cheaper and faster than nucleic acid methods and gives a better picture of fungal to bacterial ratios. Results from PLFA community analysis showed that soil pH controlled bacterial community structure well before this was found by nucleic acid-based methods [34]. It has also been suggested that phospholipid fatty acids are quickly degraded in soil, unlike DNA, so PLFA may give a better picture of the live bacterial community than DNA methods that also capture relic DNA [35].

The most important bacterial group for polyketide production is the Gram-positive phylum Actinobacteria. Within this phylum, the Actinomycetales order, have produced more pharmaceutically useful polyketides than all the rest of the bacteria [36]. This review will use both the terms Actinobacteria and Actinomycete. This is as molecular microbial ecology studies usually use the term Actinobacteria while cultivation studies typically usually use the term Actinomycetales.

The reader might think that deciding where to sample for discovery of new polyketides is straightforward. Review the microbial ecology literature to find environments where Actinomyces are diverse and a large fraction of the bacterial community. While there are a many studies that use molecular biology methods to characterize the microbial communities, using this literature to decide where to sample to find new polyketides is difficult. There are several reasons for this.

Most modern microbial ecology studies use PCR to amplify from nucleic acids extracted from the environment. The results from PCR-based methods depend significantly on the choice of extraction methods, PCR primers, and reaction conditions. Many studies use a unique version of each of these, so results are often not comparable between studies. Recently several large scale projects, such as The Earth Microbiome project [37], have been applying standardized methods on samples from across the planet.

Bacterial habitats do not follow the same pattern as the habitats of eukaryotic life that we see around us. Environments that to the human eye seem very different, can have similar bacterial communities [38]. In addition the actinobacterial community may not be controlled by the same factors as the whole bacterial community. Furthermore the Actinobacteria, which have so far supplied most of our medicinally useful polyketides, may not produce most of the type I polyketides in the environment [39]. Understanding what controls bacterial community structure and how this affects the distribution of polyketide producing bacteria is still unresolved.

Finally the major ecological question of whether bacterial distribution is controlled by biogeography has still not been settled. Biogeography, the pattern of species distribution across geographical area, affects where multicellular eukaryotes such as plants and animals are found, however it is not clear if this is true for bacteria as a whole or for polyketide producing bacteria specifically. Questions such as “Are the same bacterial communities found wherever there are the same conditions?” decide whether bioprospectors should sample particular environments in many places over the earth or many environments that may be in the same area.

We review where to sample for bacterial polyketides in terrestrial environments. The first section of this review discusses problems in using the current scientific literature for bioprospecting as briefly outlined above. Because terrestrial bioprospecting for new polyketides is already underway, the second part of this review discusses several of the terrestrial environments where new polyketides have been found or which have been suggested for bioprospecting.

The final section suggests how to sample to find new polyketides in terrestrial environments. We recommend sampling different environments and measuring samples properties in several stages. Physical/chemical properties such as pH, organic carbon or texture can be measured for a broad range of samples. The microbial community of a subset of these samples can be determined with rapid, low cost community fingerprinting, before next generation sequencing of subsets with contrasting fingerprints. An advantage of using community fingerprinting is that they give results quickly. If certain samples give unusual or promising fingerprints, the environment or area where they are from can be resampled. Bioprospectors should also try to find non-actinomycetal bacterial polyketides as they are more likely to be novel.

## 2. Unresolved Issues in Microbial Ecology that Limit Bioprospecting for New Polyketides

Molecular biological methods have changed our view of soil bacterial communities. Some Phyla, such as the Acidobacteria, are a much larger fraction of soil bacteria than was previously known [40]. Soil properties such as texture and pH are often more important in determining community structure than vegetation or climate [41]. While we know much more about bacterial communities than we did 20 years ago, there are still gaps in our knowledge that make it difficult to know where to bioprospect for new bacterial polyketides.

### 2.1. Data Comparability

**Figure 2:**
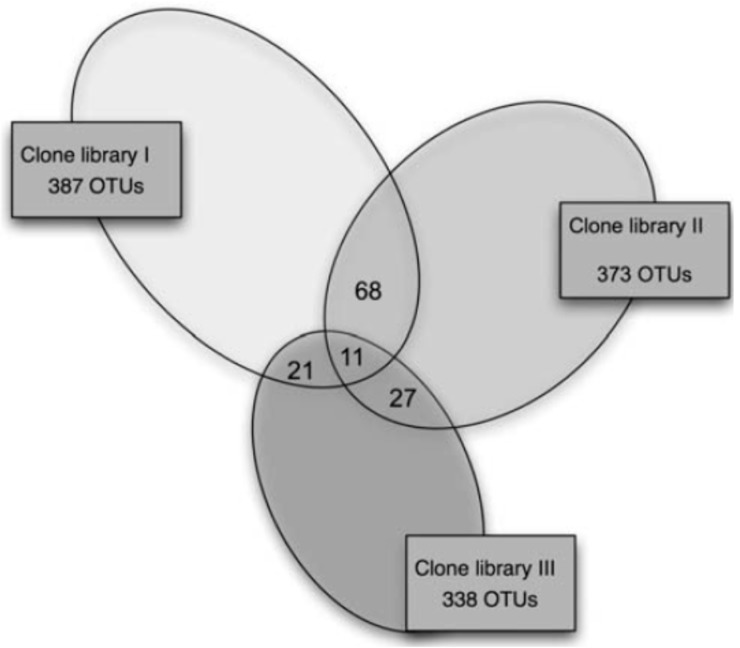
The overlap between Sanger sequencing results of three 16S clone libraries at 99% identity from a beach sand using two DNA extraction methods and two primer pairs. Clone library 1 Extraction method 1 PCR primer pair 1, Clone library II Extraction method 2 PCR primer pair 1, Clone library III II Extraction method 1 PCR primer pair 2. Reprinted by permission from Macmillan Publishers Ltd: *ISME J.* [45], copyright (2009).

Many microbial ecology studies produced data that cannot be compared with other studies. Studies often look at a single environment and look at changes in the microbial community caused by treatments, such as the effect of different tillage on soil bacteria [42], the changes in the microbial community during composting [43], or the bacteria found in different kinds of office dust. These studies extract DNA from the environment, amplify it with primers and analyze the fingerprints or sequences of the amplicons. They often use a unique combination of extraction method, primers, and amplification conditions. Changing any one of these steps can significantly change the results. For example, Delmont et al. [44] compared a range of DNA extraction methods on two grassland soils and found that they gave very different pictures of the microbial community. Hong et al. [45] used two extraction methods and two primer sets to measure the bacterial community of a beach sand sample, only 11 of 1098 operational taxonomic units (OTUs, defined as 16S ribosomal RNA genes that have less than 99% similarity) sequences were found in all three data sets (Figure 2). In our own work two sets of “actinobacterial specific” and one set of eubacterial 16S primers amplified very different groups of the actinobacteria [46]. Even when the same methods are carried out in different laboratories there can be significant differences in results [47].

Bioprospectors need to know where to sample. The most useful results will come from studies that use the same method over a broad range of samples from different environments. As methods of analysis are continually improving, the samples (along with information about where and when they were collected and what they are like) should be stored for reanalysis as new methods come online [48]. Several large collaborative projects have been set up to do exactly this [37,49,50,51], and this is an area of research that is expanding [52].

These consortia have the choice of using methods that are labour and time intensive but give a lot of information for a few samples or using methods that are faster and cheaper, but less detailed for many samples. The best example of the time and labour intensive approach is the Terragenome project [49]. This consortium began by sequencing the metagenome of a few well characterized soil samples, starting with samples from the oldest field trial in the world at Rothamsted. Eventually this work will provide very detailed information on polyketide content in a few soils.

Shotgun metagenome sequencing of the diverse microbial communities from soil is difficult. An enormous volume of sequence data is needed and it is difficult to assemble genomes or polyketide biosynthetic gene clusters from these large data sets [53]. Terragenome involves investing a lot of time, work and funds in a few soils before it is known how representative their microbial communities are. It has been suggested that it would be more efficient to survey many soils using simple 16S bacterial community fingerprinting methods, before picking samples for direct metagenomics sequencing [54]. This initial step has been done in soil microbial surveys of Great Britain and France, where hundreds of samples from across landscapes have been fingerprinted [55,56]. These studies provide reproducible but low resolution information on bacterial distribution and diversity in soil.

The most ambitious of these projects, which uses identical molecular methods on many samples, is the Earth Microbiome project. This project samples broadly from environments including soil, marine sediment, and the digestive system of mammals. It uses next generation sequencing to sequence 16S bacterial and 18S fungal ribosomal RNA amplicons, as well as shotgun metagenome sequencing. Recently a host of additional projects looking at the microbiome of humans have used next generation sequencing of 16S amplicons to characterize thousands of samples [57,58]. These studies are beginning to provide a clearer picture of bacterial community structure of many environments.

The laboratory of Brady, a pioneer in polyketide bioprospecting, has developed a database, the Environmental Surveyor of Natural Product Diversity (eSNaPD) that includes information on the distribution of polyketide synthases in the environment. So far it is limited to soils and marine sediments from the Southwestern US and New England [59].

### 2.2. Soil Bacterial Habitats Do Not Correspond to Eukaryotic Habitats

For over a hundred years, terrestrial ecologists have studied how eukaryotic life on Earth is distributed. Plant and animal life is distributed over the Earth in biomes, such as tundra, rainforest, prairie, etc. These biomes are more diverse in the tropics and diversity decreases with distance from the equator [60]. When biomes such as tropical rainforest are converted to crops or pasture [61], biological diversity decreases. Given what we know about these biomes, it would seem reasonable to bioprospect for new polyketide natural products in diverse tropical biomes undisturbed by human activity. However while fungal distribution may follow these biomes [62], bacterial distribution does not.

The plants that grow on soil are probably not the best guide to the bacteria that live in them. Soil properties better predicted bacterial community structure than vegetation in several early studies that looked at soils and land uses [63,64,65]. In 2006, a study by Fierer compared 88 soils under natural vegetation from the Peruvian Amazon and across the continental United States [66]. Soil DNA was used for 16S community fingerprinting (T-RFLP). Fingerprints from soils close to each other but of different pH differed but resembled those from thousands of miles away with the same pH. Soil pH also determined how diverse the fingerprints were, with the most diversity at neutral pH. This effect of pH on bacteria community structure has been confirmed by studies that compare soils from narrower geographical regions such as the Arctic [38] and Malaysia [41].

The importance of pH to bacterial community structure leads to non-intuitive conclusions for microbial bioprospectors. Biomes such as tundra may have similar soil bacterial communities to biomes with far more and diverse vegetation [38]. Tropical rainforest soil, underneath one of the most biodiverse plant and animal ecosystems on earth, has a relatively undiverse bacterial community compared to soils from ecosystems that have orders of magnitude fewer species of plants and are thousands of miles further north [66]. Converting Amazonian tropical rainforest to cropland or plantation may increase soil bacterial diversity [67].

While pH may be the major controlling factor for soil bacterial community structure, it is not the only one. When comparing soils over a narrower range of pH, other soil properties are important. A study in The Netherlands compares 25 sites over a broad range of soils and land uses. When three acid pine forest soils were excluded (pH 3.7–4.1), soil phosphorous controlled bacterial community structure in the remaining soils (pH 5.1–7.6) [68]. On a range of potato soils in Germany of similar pH (5.2–6.2), parent material controlled bacterial community structure [69].

Microbial bioprospectors cannot rely on the vegetation that they see around them to know where to sample soil. The will need to know a range of soil properties that can be readily determined in the laboratory or through soil surveying. Unfortunately many studies of both bacterial and polyketide distribution in soil do not include soil properties.

Eukaryotic and bacterial communities do not just differ in how they are distributed, bacterial communities are far more diverse. Fierer and Lennon discuss why this is, two of the most important reasons being phylogenetic breadth and geographical scale [70]. Bacteria are one of the three domains of life so a fair comparison of the bacterial diversity of soil would be with the diversity of all of the eukaryotes living in the soil (i.e., plants, animals, fungi, protozoa etc.). More important for bioprospectors is the question of scale. Grasses are sampled in 1 m square plots which can have 10,000 individual grasses, to sample on this scale for bacteria microbiologists would have to sample a single fine sand grain. To sample grasses on the scale that we sample bacteria (tens to hundreds of billions) plots would have to be 10 km^2^. Grass in a 10 km^2^ area would have differences in their environment, the same would be even truer of bacteria in soil, a complex environment, where there are changes in conditions at the µm scale.

### 2.3. Actinobacterial Soil Community Structure May Not Follow Bacterial Soil Community Structure

**Figure 3:**
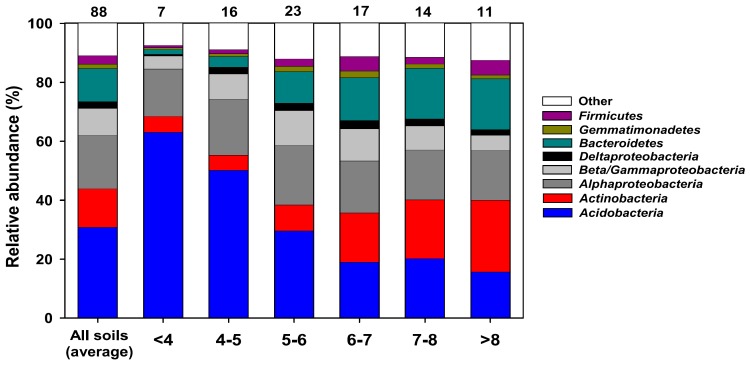
Bacterial 16S 454 pyrosequencing results from 88 non-agricultural soils from across North and South America. Reproduced with permission from Lauber et al., *Appl. Environ. Microbiol.*, published by the American Society for Microbiology, 2009 [71].

Actinobacteria cultured from soils are the traditional source of new polyketides. A follow up to the Fierer study, which found that the soil bacterial community structure was controlled by soil pH [26], used next generation sequencing of 16S amplicons obtained from their original samples and found that Actinobacteria were most common at higher pH [71] (Figure 3). The actinobacterial fraction of the bacteria increased from 5.35% at pH < 4 to 24.3% at pH > 8 but actinobacterial diversity was greatest at an intermediate pH between 6 and 7. The same relationship between the relative abundance of Actinobacteria and pH was found for the bacterial communities of polar soils [38]. This suggests that neutral to alkaline soils may be the best target for actinobacterial bioprospecting.

However actinobacterial abundance and distribution may not be controlled by soil pH in the same way that the whole bacterial community is. At the continental scale soils in dry climates are neutral to alkaline and soils in wet climates are acidic. At the landscape level soil parent material can determine soil pH. Desert soils are enriched in Actinobacteria compared to soils from wetter climates [72]. Field plot studies where the pH of a plot of soil is adjusted over a gradient can separate the effect of pH from climate. Four field plot studies have been carried out on cropped [49,73] and pasture soils [74,75] in the United Kingdom. While they have found that the actinobacterial community structure changes with pH [74,75,76], they have not found that the relative abundance of the actinobacterial fraction depends on pH through sequencing of 16S amplicons [46,47,49] and phospholipid fatty acid abundance [48]. In two of these cases the relative abundance of other major bacterial phylogenetic groups changed [46,47], in another there was no change for any of the bacterial phyla, although there were changes at lower phylogenetic levels [49]. These results suggest that actinobacterial relative abundance is not controlled by pH but do not say what does control it.

**Figure 4:**
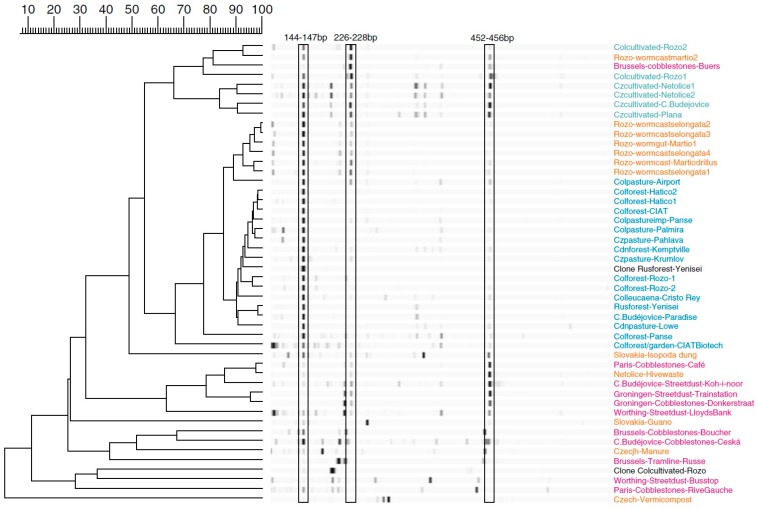
Forward Terminal Restriction Fragment Length Polymorphism (T-RFLP) community fingerprints of the Actinobacteria from Uncultivated soil (blue) Cultivated soils (green), animal associated sediments (orange) and street dust (red). Reproduced from Hill et al., *Microb. Ecol.*, published by Springer International Publishing AG., 2011 [46].

All of the above studies have either compared soils under natural vegetation or looked at a single agricultural land use (cropping/pasture). Converting native vegetation to agricultural use often increases the actinobacterial fraction of the soil bacterial community [65,77], in particular when humid tropical forest is converted to crops [67,78] although this effect can be below statistical significance [79]. This may be because under native forest these soils are acidic and deforestation raises the soil pH slightly. This is the explanation given for the effect of land use (cultivation, pasture, pine forest, and deciduous forest) on soil bacterial community structure in a well cited paper by the Fierer group. The authors examined twelve sites in South Carolina and it was shown that land use changed the bacterial community by changing pH rather than any other inherent effect of the land use [77]. However the relative abundance of Actinobacteria can increase as pH decreases during conversion of forested land to cropping [80]. In our own study of contrasting soils from Colombia, Canada and Europe using community fingerprinting with two sets of actinobacterial 16S primers, we found that fingerprints clustered by land use (cultivated versus uncultivated), with no effect of pH [46] (Figure 4).

There are several ways that land use could affect the relative abundance and community structure of soil Actinobacteria. The higher level of Actinobacteria in cultivated and pasture lands versus forest in Alabama [81] or palm oil plantation versus forest in Borneo, Malaysia [82], led researchers to suggest that Actinobacteria are selected by human disturbance. A study in the savanna region of Ghana found that soils with no or little vegetation between corn cropping seasons had a higher relative actinobacterial fraction [83] than soils with more vegetation.

Land use can also affect the bacterial community structure by adding or removing substrate for bacterial growth. Bacteria can be classified as oligotrophs or copiotrophs, similar to K- and r-selected plants and animals. Oligotrophs have an advantage when food is limited and grow slowly. Copiotrophs are more competitive when food is plentiful. Fierer et al. measured carbon and nitrogen mineralization in 71 soils and compared this with the relative percentage of the Actinobacteria and five other bacterial groups, α-Proteobacteria, β-Proteobacteria, Acidobacteria, Bacteroidetes and Firmicutes, as measured by quantitative real-time PCR. They concluded that while the Acidobacteria were oligotrophs and the Bacteroidetes and β-Proteobacteria were copiotrophs, the α-Proteobacteria, Firmicutes and Actinobacteria were neither [84].

Whether Actinobacteria are oligotrophic or copiotrophic may depend on the nature of the substrate. Additions of carbon rich straw or reduced tillage that leaves straw on the surface of the soil reduces the relative amount of Actinobacteria in the soil bacterial community [42]. Adding nitrogen fertilizer increases the actinobacterial fraction of the soil bacteria. An increase in the Actinobacteria with nitrogen was found for 28 soils from natural vegetation across the continental United States by next generation sequencing of 16S amplicons [85], as well as at a grassland and cultivated corn site through next generation sequencing of 16S amplicons [86,87] and shotgun metagenome sequencing [60]. Both nitrogen and potassium addition increased the actinobacterial fraction of a Dutch grassland soil as measured by shotgun metagenome sequencing [88].

A 100 year old field trial in Alabama compared the effect of adding lime to raise pH of an acid soil versus adding lime and fertilizer. The bacterial community structure was measured using PLFA analysis. While pH had more of an effect on the overall bacterial community structure than nitrogen fertilizer, nitrogen fertilizer had a major effect on the actinobacterial and fungal levels [89]. An increase in the actinobacterial fraction of the bacterial population has been ascribed to the flush of nutrients produced by burning after deforestation in the Amazon [51].

More work is needed comparing the relative importance of pH, moisture, land use and nitrogen on actinobacterial community structure and relative abundance in soil. There may not be a simple relation between these factors. Just as the actinobacterial community structure does not follow that of the whole bacterial community, different sub groups of the Actinobacteria may be selected for by different factors. For example, Actinobacteria can be the largest fraction of the bacterial communities of deserts [72] and lake waters [90,91]; probably different mechanisms of selection are occurring in these cases. This uncertainty means bioprospectors need to compare a broad range of soils of differing properties, land uses, and nutrient inputs.

### 2.4. Non-Actinobacterial Bacterial Polyketide Producers

While most polyketides discovered by cultivation of bacteria in the laboratory so far have come from Actinobacteria, many bacterial polyketide producers in the environment are not Actinobacteria. In the case of aromatic type II polyketides most producers are likely Actinobacteria. Genes encoding type II polyketide synthases (PKSII) amplified from the soils DNA either directly [92,93,94,95] or from metagenomic libraries [96] appear to be entirely actinomycetal. The only example where PKSII amplicons appear to be not-actinomycetal is a set of five out of fifty-seven sequences amplified from European soils. In this case the PKSII amplicons appear to be from proteobacteria or firmicutes [96].

In contrast many studies have found environmental amplicons and clones with type I polyketide synthase (PKSI) sequences that cluster with non-actinobacterial sequences. A broad range of studies have used PKSI specific primers to amplify soil DNA followed by cloning and Sanger sequencing. For example, most PKSI sequences from soils from Malaysian rainforest [94], an Antarctic Island [97,98], close to cucumber roots [99], and the Tibetan plateau [100] clustered with non-actinobacterial sequences when treed.

Three studies have sequenced metagenomics PKSI clones from soil that do not appear to be actinomycetal. As part of the European Union funded Metacontrol project, a metagenomic library was made of environmental DNA from a French cultivated soil [101]. The 60,000 clones in the library were screened with PKSI specific primers and 139 positive clones found. Three of these clones were fully sequenced. Two PKS domains from these clones, the ketosynthase (KS) and acyltransferase (AT) domains clustered with myxobacterial KS and AT amino acid sequences. A fosmid library from a cultivated soil in the Metagenomic project at the [39] University of Wisconsin, was screened and 29 clones were identified as containing KS domains by hybridization. The inserts of five of these clones were sequenced. Their KS domain homology suggested that they were from the Proteobacteria or Cyanobacteria. However analysis of the full insert, its GC content, and comparison with KS domain sequences from three acidobacterial isolates suggested that they were from the Acidobacteria.

As a final example, DNA was extracted from a Brazilian eucalyptus plantation soil, and made into a fosmid clone library. A clone with a PKSI hit was sequenced [102]. The start of the clone contained a single PKSI module with 20 other open reading frames (ORFs). Matches to the ORF were all non-actinomycetal, while the KS and AT domains of the PKSI module clustered away from their actinobacterial counterparts when treed with the SEARCHPKS database [103]. This database was disproportionately actinomycetal possibly biasing the analysis. However this result was confirmed when the sequences were reanalyzed with a broader range of non-actinomycetal PKS domains [104].

These environmental PKSI sequences were probably non-actinomycetal, but as only part of their genome was sequenced we cannot be certain which bacterial group they were from. In some environments most PKSI producers are actinomycetal.

In our work we compared PKSI amplicons of KS and AT from street sediments [104], which are enriched with Actinomycetes, and soils [46]. Many soil amplicons clustered with myxobacterial sequences. However several clades clustered with actinomycetal sequences and were specific to samples from the streets of Ottawa (Canada), Faisalabad (Pakistan), and four European cities. The Brady group compared PKSI KS sequences in soils from New England and desert soils of the Southwestern United States [105] through pyrosequencing amplicons. KS domain diversity was greater in the actinobacterial rich desert soils.

As further evidence that non-actinomycetal source may prove important in bioprospecting, a new class of antibiotics (albeit a non-ribosomal peptide natural product) was recently discovered through the use of an in situ cultivation technique that grew a novel β-proteobacteria from soil [17]. Non-actinomycetal producing organisms may be important for future polyketide discovery.

### 2.5. The Question of Biogeography

The term biogeography is used in two ways in microbial ecology. Often it simply means changes in the community over geographical distance. An example of this is the study that found that the actinobacterial and PKSII communities of soils in Uzbekistan and New Jersey differed [93]. Biogeography in this sense was discussed in the earlier section on the importance of pH and other soil properties in the distribution of bacteria and actinobacteria (see Section 2.2 and Section 2.3). In the narrower sense, used in this review, biogeography means that communities in similar environments that are separated by distance or a barrier such as an ocean will have different microbial communities. This difference is attributed to the communities evolving separately as Wallace proposed in the 1876 *Geographic Distribution of Animals* [106]. Biogeography can have a large effect on the distribution of large multicellular organisms. For example, marsupials are found in Australia but not in Africa, even though there are similar environments in both places.

For polyketide discovery, the more polyketide synthase gene distribution in terrestrial environments is controlled by biogeography, the more important it is to sample similar habitats that are separated from each other by distance or geographical barriers. If biogeography is not important, it is more important to sample different habitats, even if they are close to each other. Addressing this question is complicated by the difficulty of defining bacterial, actinobacterial and polyketide habitats.

Microbes face fewer barriers to distribution than larger organisms. It has been argued that there are no barriers to organisms <20 µm because they are easily spread across the globe and their numbers are so high [107]. This is a restatement of ”*Everything is everywhere but the environment selects*”—a famous quote from the first half of the 20th century. There is disagreement as to who is being quoted and what was said [108] and some maintain that this view has roots in the 19th century [109] but it stems from the observation that the bacteria isolated depended more on the isolation method than the sample. If this is true, bioprospecting depends on developing culturing methods that favour the growth of novel microbes “*perhaps using virtually any natural sample*” [110].

In the 2000s, as molecular methods were introduced, the question of microbial biogeography was revisited [111]. This was partly because the it was shown that isolated bacteria were “weedy” (i.e., found everywhere and fast growing) compared to the uncultivated majority of bacteria that could only be studied with molecular methods [112].

A clear biogeographical effect was found for archaea [113] cyanobacteria [114] and actinobacteria [115] from volcanic hot springs that are thousands of miles apart. However hot springs are isolated from each other so this does not mean that biogeography is important in broadly distributed environments like soil. This is an argument for ensuring that cave microorganisms, which are also in highly isolated, are protected from contamination by cavers and scientists [116].

National level surveys of the soils of France [56] and Britain [55] using fingerprinting have found little or no biogeographical effect. This may be because it is difficult to find identical soils that are distant from each other at the national scale [55].

Several studies, which have used the higher resolution methods of next generation sequencing, have looked for biogeographic effects on bacteria in soil. A comparison of mollisols (soils developed under prairie) of Manchuria found that environment rather than distance had the greatest effect on bacterial community structure, although distance may have had some effect [117]. A biogeographic effect was found for Actinobacteria in soils that were recently been exposed by receding glaciers [118]. This effect was not found for Chinese glaciers in a study that used the same methods of DNA extraction and amplification, but was observed when they were compared with literature data from other continents. As different methods of PCR were used for some of the literature samples, this result may be an artefact of the method.

A novel approach to the question of soil bacterial biogeography was to transplant soils from two sites in China around 1000 km apart, leave them for 20 years, and then use next generation sequencing to see if their bacterial communities were the same as in the soils that they had come from or their “new homes”. After 20 years the bacterial communities resembled those of the soils around them; from this the authors argue against a biogeographical effect [119].

Five research groups have compared polyketide distribution in soils sampled far from each other and three have reported evidence of biogeography. However in two of these cases, this may be due to sampling different habitats rather than geographical separation.

Wawrik et al. compared actinobacterial and PKSII T-RFLP fingerprints from an acid New Jersey Pine forest soils and a range of undefined soils from Uzbekistan and concluded that there was a biogeographical effect [93]. Given that the climate, soil pH, soil type, and land use of New Jersey and Uzbekistan is very different, this is not surprising.

Morlon et al. amplified part of type II αKS domains from three sites under Mediterranean vegetation in South Africa, Australia and Chile [95]. While the vegetation in all three areas may have been similar it is not clear that the soils were. The landscape varied between a flat plain (Australia), rolling hills (Chile) and along a ridge (South Africa). While they identified a large clade of αKS domains found in all three sites, their overall advice for bioprospectors was to sample broadly on a range of different continents.

**Figure 5:**
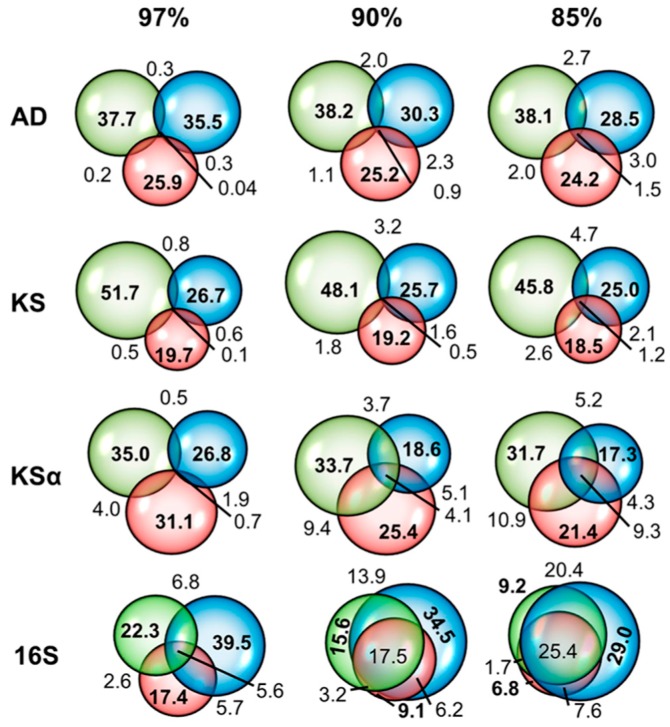
Overlap of sequences amplified from three soils from the desert sites in Arizona (green), Utah (blue) and California (red). Sequence are from non-ribosomal polypeptide (NRP) Adenylation (AD) domains, type I polyketide synthase PKS ketosynthase domains (KS), type II PKS alpha ketosynthase domains (KSα) and 16S ribosomal sub units. Adapted with permission from Reddy et al., *Appl. Environ. Microbiol.*, published by American Society for Microbiology, 2012 [120].

The strongest arguments for biogeography have come from the Brady laboratory who have identified biogeographic effects between three soils in the arid deserts of the Southwestern United States [120], between 96 samples from these deserts and New England [105] (Figure 5) and among a collection of these 96 American samples and an additional 96 samples from China, Brazil, Alaska, Hawaii, Costa Rica, Ecuador, the Dominican Republic, Australia, Tanzania and South Africa [121].

In this last publication samples were characterized by next generation sequencing of Adenylation (AD) domains from non-ribosomal polyketide (NRP) pathways and KS domains from modular PKSI pathways. There was strong evidence of biogeography as location was the most important factor in determining sequence composition. The samples that were most similar to each other were geographically close to each other, even though they were often from different biomes. Similar biomes on different continents had less than three percent of sequences in common. Within samples that were close to each other, samples of similar biomes were most similar to each other. Certain pathways were much more common in particular samples. Two areas, the deserts of the American South West and the Brazilian Atlantic forest, had soils where there was an order of magnitude more KS and AD diversity than other soils, making them attractive targets for bioprospecting.

Two papers present evidence against a biogeographic distribution of polyketide synthase genes. As part of a study that describes a screening method for PKSII and NRP sequences in metagenomics libraries, libraries of environmental DNA from Antarctica, Cuba and Europe were screened and clones sequenced [96]. No evidence was found of clustering by area.

Our laboratory extracted DNA from soils and street sediments in the Americas and Europe. DNA was amplified with PKSI specific primers before cloning and sanger sequencing [104]. Sequencing was extremely shallow and broad, with between three and 18 sequences for 21 samples. Several clusters of sequences were found in many sites thousands of miles from each other. In some cases these clusters were from street samples (e.g., Ottawa and Budapest) where human activity might be expected to carry and mix Actinomycetes from around the world. However a group of sequences that appeared to be myxobacterial was found in soils from the Canadian Arctic and Europe. While there were differences in sequences from site to site, these differences were comparable to those found between highly similar known pathways. Even if they are biogeographical effects in sequence composition, they may not be enough to affect polyketide function.

It does not matter to bioprospectors whether or not biogeography exists. What matters is if or how much it inconveniences sampling. Two recent studies of the microbiology of New York parks suggest that biogeography may not be an obstacle to bioprospecting. These studies used next generation sequencing to characterize either ribosomal or PKSI amplicons.

The ribosomal study, conducted by the Fierer group, extensively sampled Central Park (40,000 sequences/sample, 596 samples) [122]. Bacterial community structure was found to be controlled by pH, and varied greatly over the park, probably because of a wide range of soil management. Sequences from 52 of these samples were randomly picked and compared with 16S sequences from 52 soils (also 40,000 sequences/sample) taken from a broad range of environments in Canada, the United States, Peru, Argentina and Antarctica [72]. There was considerable overlap between the two datasets. 94% of the most common sequences were found in both and the diversity (as measured in rarefaction curves) was only slightly less in the central park dataset. The few sequences that were not found in Central Park were from deserts. The authors concluded that distance and climate is relatively unimportant in determining bacterial community structure.

A study carried out by the Brady group took 275 soil samples from 41 parks in the New York City area, amplified extracted DNA with type I polyketide synthase (PKSI) and non-ribosomal peptide (NRP) primers, and sequenced the amplicons with next generation sequencing. NRP amplicons were compared to similar amplicons from 96 soil samples from four areas of the continental United States, and they clustered apart, suggesting that they were New York City specific sequences. However sequences encoding the biosynthesis of 11 known polyketide and non-ribosomal peptide medicines that had been initially discovered all over the world, could be found in one or more of the New York City samples [123]. The Brady group suggested that the same might be true of many samples and suggest that bioprospectors look deeply in a few samples rather than “*scratching the surface*” of many. They note that in some cases natural products originally found in marine microorganisms, can be found in terrestrial environments.

It could be argued that these results do not disprove the existence of bacterial biogeography as a large city will have many introduced bacteria from around the world and human activity will provide a broader range of substrates for them to grow. These effects have been found for earthworms in Australian cities [124] and fungi in Vienna, Austria [125]. In a practical sense, for polyketide bioprospecting, this biogeography will not matter as sampling large cities is relatively easy compared to sampling many soils from around the world. Biogeography nevertheless remains an important unsettled question in bioprospecting. A recent (2017) review of metagenomic antibiotic bioprospecting in soil considered that biogeographic studies would probably be essential to finding new antibiotics [126].

## 3. Bioprospecting for New Polyketide Discovery

Uncertainty about how polyketide synthase genes are distributed in the environment has not stopped bioprospecting. Most previous polyketide discovery has been through isolating Actinomycetes from soil. As fewer new polyketide are discovered this way, bioprospectors have begun to look at alternatives ways of sampling soil as well as alternatives to soil. The most common alternative to terrestrial soil is marine environments, a topic covered by many other reviews (e.g., [127]). Here we will review some recent examples of bioprospecting from terrestrial samples, including insect associated bacteria, desert soils, and disease suppressive soils. We will also discuss environments that might be good targets for bioprospecting.

### 3.1. Terrestrial Environments Where Polyketides Bioprospecting Is Underway

#### 3.1.1. Eukaryotic Associated Bacteria

One of the more promising environments for bioprospecting is the bacteria that are associated with or symbiotic to terrestrial eukaryotes. Jensen and Fenical in 1996 recommended bioprospecting from bacteria that live in close association with marine plants and animals, a strategy that many in natural product discovery community have since followed. Jensen and Fenical’s rationale applies equally well to terrestrial macrobes, such as insects. Bacteria that are sheltered or in association with multicellular organisms inhabit environments that vary both between and within individual eukaryotic species, leading to innumerable, highly specific microenvironments. As the bacteria-host interaction becomes more complex, there is more chance of finding bacteria that have adapted to the specific environment. Furthermore the probability of finding that adaptation outside of the interaction is highly unlikely. Thus these bacteria-host interactions offer a highly unique environment with distinct and unique bacteria taxa. If these environmental adaptations affect polyketide production, then these bacteria represent an enormous potential for new polyketide discovery [128]. As well as this ecological argument for exploiting eukaryotically associated bacteria, we now know that many secondary metabolites once thought to be produced by eukaryotes are now known to be made by their associated bacteria and that the bacteria and their eukaryotic hosts have coevolved [129,130]. In particular, *Streptomycetes* are proposed as a phylogenetic group with a long evolutionary history of symbiosis [131].

##### Insect-Associated Bacteria

The most studied polyketide producing bacteria associated with Eukaryotes are those found on insects. Insects are heavily colonized by microorganisms with between 1% and 10% of their biomass being microbial so it is not surprising that insects and microbes have developed mutualistic interactions. Kaltenpoth reviews mutualism between Actinomycetes and insects [132]. Insects have mutualistic relationships with bacteria for nutrition but Actinobacteria are rarely involved in this (see [133]). Instead actinobacterial mutualism is usually defensive. These defensive interactions can be difficult to study. Defensive bacteria may not be on insects in large numbers for their full life cycle. Often they are found when the insects are in an immobile life stage such as eggs or larvae and so vulnerable to infection or predation. Nutritional mutualistic bacteria are vertically transmitted (i.e., transmitted from parent to offspring), defensive bacteria however may be acquired from the environment [134].

There may be many undiscovered non-obligatory symbioses, particularly among fungus farming insects and insects that live in soil or rotting wood where there is continual contact with bacteria [104]. Both ants and bark beetles farm fungi, and Actinomycetes protect the farmed fungus from infection (discussed below). Actinomycetes make good defensive symbionts not only because the produce secondary metabolites but also because they can use a wide variety of substrates for growth including eukaryote excretions. In addition actinomycetal spores are easily transmitted between insects. Fungus farming bees have also recently been discovered [135] and unidentified Actinomyctes and their natural products may similarly protect them. In this section we will review social and non-social insect associated bacteria and their polyketides. Several other publications review this topic more extensively [136,137,138,139].

##### Social Insects as Sources for Bacterial Polyketide Discovery

**Figure 6:**
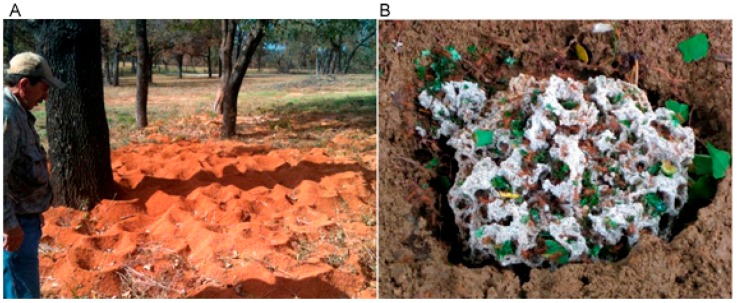
(**A**) Leaf cutter ant colonies in Texas. Photo courtesy of Texas A&M AgriLife Extension/Josh Blanek; (**B**) Leaf cutting ant nest in Costa Rica. In this case the nest was exposed when a rain barrel was moved, normally they are found at greater depth. Photograph courtesy of Herster Barres, *Reforest the Tropics*.

Social insects are a promising source of new polyketides. Lombardo suggests that social behaviour is driven by the need to keep and coevolve with microbial symbionts [140]. Crowding together helps symbionts that can defend their host pass between insects. The best known social insects with antibiotic producing Actinobacteria are the attine ants (Figure 6). Debates over the relationship between attine ants and their Actinobacteria illustrate issues involved in bioprospecting bacterial symbionts.

Many ants cultivate fungus for food. In primitive forms of ant fungal agriculture, ants collect debris to feed fungi. The most developed form of this symbiosis is that of leaf cutting ants who grow fungus (genus Leucoagaricus) on leaf fragments that the ants provide. This fungus grows in underground gardens that the ants maintain (Figure 6B). These fungal gardens can be infected by pathogens, such as the fungus *Escovopsis* (Ascomycota: anamorphic Hypocreales). The ants, the fungus and the fungal pathogen appear to have coevolved [141].

In 1999 Currie et al. [142] described a fourth part of this system. The bodies of these ants are covered in a whitish gray material originally thought to be exuded by the ants themselves. In a survey of 22 species of leaf cutting ants from Panama and Ecuador, Currie et al. identified this white material as Actinomycete biomass. This Streptomyces was vertically transmitted (i.e., down generations) and found in all species of attine ants. These Actinomycetes were tested for antagonism against several strains of fungi. There was little inhibitory effect on generalist fungi, but they did inhibit *Escovopsis*, which infects attine fungal gardens. The Actinomycetes strain also encouraged the growth of the fungi that the ants were farming. The system was a complex quadripartite symbiosis between ant, fungus, pathogen and Actinomycete, in which the ant, fungus and Actinomycete are in an arms race against the fungal pathogen [141].

Later work showed that the Actinomycete in question was *Pseudonocardia* rather than a *Streptomycetes*. More importantly, evidence emerged that suggested that *Pseudonocardia* were picked up from the environment rather than passed down through generations. When leaf cutting ants were raised in several laboratories, 16S sequences from *Pseudonorcardia* on them clustered by laboratory rather than ant species [143]. Secretions from the *Pseudonocardia* were more antagonistic to the farmed fungi than the infecting *Escovopsis*. It was suggested that the *Pseudonocardia’s* role may not be to protect the farmed fungi but the ants themselves or the ant nest and that there may not even be a mutualistic relationship [144]. Since then more evidence has been found suggesting that *Pseudonocardia* have coevolved with attine ants and *Escovopsis*. A comparison of colonies in Costa Rica and Panama found that pseudonocardial distribution followed that of *Escovopsis* [145]. Recently, evidence of lateral transfer of polyketide synthase genes between strains of *Pseudonocardia* has been found [146].

It is beyond the scope of this review to say whether leaf cutting ants get their Actinomycetes from their ancestors, other leaf cutting ants, or the environment (see [116,117,118,119,147]). There are implications for sampling that apply to all cases where there are these alternatives. If polyketide producing Actinomycetes in eukaryotes are acquired vertically, bioprospectors should sample by eukaryote phylogeny. If polyketide producing Actinomycetes are continually acquired from the environment, bioprospectors should view ant nests as an environment that is enriched in bacterial secondary metabolite producers.

**Figure 7:**
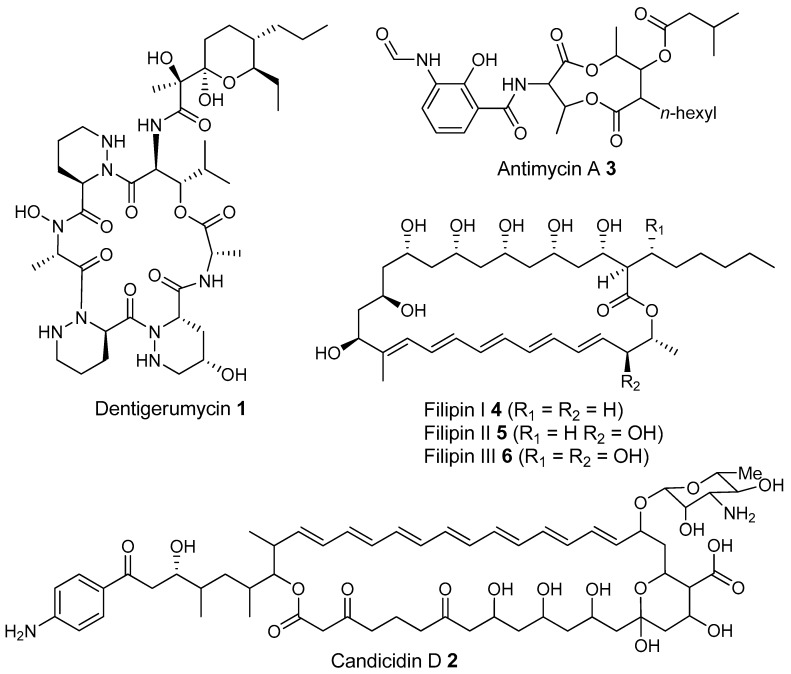
Polyketide and non-ribosomal peptide natural products isolated from bacteria associated with social ants.

Most of the work on antibiotic production by attine associated bacteria has been done on Actinomycetes (e.g., [148]) although β-proteobacteria have also been found in fungal gardens with antifungal activity specific to fungal pathogens [149]. Both known and novel polyketides have been found to be produced by attine ant associated Actinomycetes (Figure 7). Dentigerumycin, **1**, is a mixed non-ribosomal peptide type I polyketide with a novel core structure that was produced by a *Pseudonocardia*. The pseudonocardial strain was isolated from the cuticle of the attine ant species *Apterostigma dentigerum* from Panama. Dentigerumycin has [150] antifungal activity against *Escovopsis* and also inhibits several strains of Candida.

*Pseudonocardia* is not the only ant-associated bacterium producing polyketides. A *Streptomyces* from a fungal garden of a Panamanian ants nest produced candicidin (**2**) [148]. The *Streptomycetes* strain that produces this polyene antibiotic has also been also been isolated from ants’ nests in Trinidad [151].

The study that identified the candicidin producing *Streptomyces* in a Trinidadian ant nest was part of a pair of publications. The first isolated a number of *Streptomycetes* and *Pseudonocardia* from the nests and found that the *Streptomyces* (strain S4) produced candicidin. The *Pseudonocardia* was shown to produce an analog of nystatin called nystatin P1. While the complete structure of nystatin P1 was not solved, both the biosynthetic machinery, deduced from the genome sequence, and liquid chromatography–mass spectroscopy (LC–MS) analysis of the culture media are consistent with a nystatin A_1_ derivative possessing an additional dideoxy amino hexose [123].

A following study sequenced the genome of the *Streptomyces* S4 strain [152]. The biosynthetic gene cluster encoding candicidin was identified in the genome as well as a number of other uncharacterized biosynthetic gene clusters. The authors were able to assign one of these gene clusters to the known mixed polyketide-non-ribosomal peptide antimycin (**3**). When the candicidin and antimycin gene clusters were disrupted in *Streptomyces* S4, it was still active against *Escovopsis*. This suggests that additional natural products encoded by the many uncharacterized gene clusters in this strain may be responsible for the antibacterial or antifungal activity. Isolation and characterization of these compounds is still unaddressed.

Recently a polyene was discovered on two strains of *Pseudonocardia* in the La Selva biological station in Costa Rica. Selvamicin was found to have less antifungal potency but was more soluble than the similar nystatin. The genes for selvamicin production were found on the genome of one pseudonocardial strain and in a plasmid on the other. The genes were more similar to each other than they were to the genomes of either *Pseudonocardia*, suggesting lateral gene transfer [146].

Social ants do not only use fungi for food. Gao et al. describe the use of fungi by *Allomerus* ants to build traps for insects on plants [153] and found using activity guided fractionation and LCMS-based comparisons to authentic standards that Streptomycetal isolates from *Allomerus* ant bodies produced the filipins **4**–**6**, a family of type I polyketides also produced by *Streptomyces. avermitilis*.

While fungus growing attine ants are the main insect herbivore in the tropics of the Americas, in tropical sub-Saharan Africa and Southeast Asia this role is taken by fungus growing termites. Their ecological roles are similar. Unlike the attine ants, the fungi that termites eat (a Basidiomycetes of the genus *Termitomyces*) appear to spread laterally rather than vertically [154]. As with the attine ants, unwanted fungi can compete with the farmed fungi. In particular, the Ascomycete *Pseudolxylaria* appears to compete with *Termitomyces* for food. As with the ants, Actinobacteria likely play a role in combating these pathogens. A study which cultured Actinobacteria from 30 South African termite nests of three genera of termites found that while the Actinobacteria (*Streptomycetes, Micromonospora*, and *Actinomadura*) often had anti-fungal activity, they were more likely to inhibit the *Termitomyces* than the *Pseudolxylaria*. There was no evidence of Actinobacteria clustering by termite genera and their closest 16S matches were often to Actinomyetes isolated from other insects such as the Southern Pine beetle [155], suggesting that the Actinobacteria in the termite four way relationship were acquired from the environment rather than vertically transmitted.

**Figure 8:**
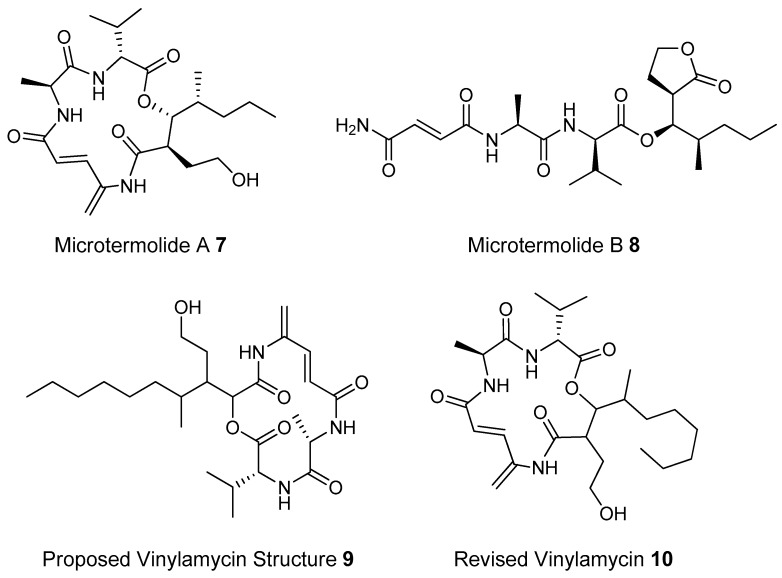
Microtermolides, produced by termite-associated microbes, and the originally proposed and revised structure of the related NRP vinylamycin.

Potent and often structurally unique polyketides can be produced under these conditions. A Streptomycete strain isolated from South African termites was cultivated and found to produce two related hybrid non-ribosomal peptide polyketide derived compounds, microtermolides A and B (**7** and **8**, respectively, Figure 8) [127].

**Figure 9:**
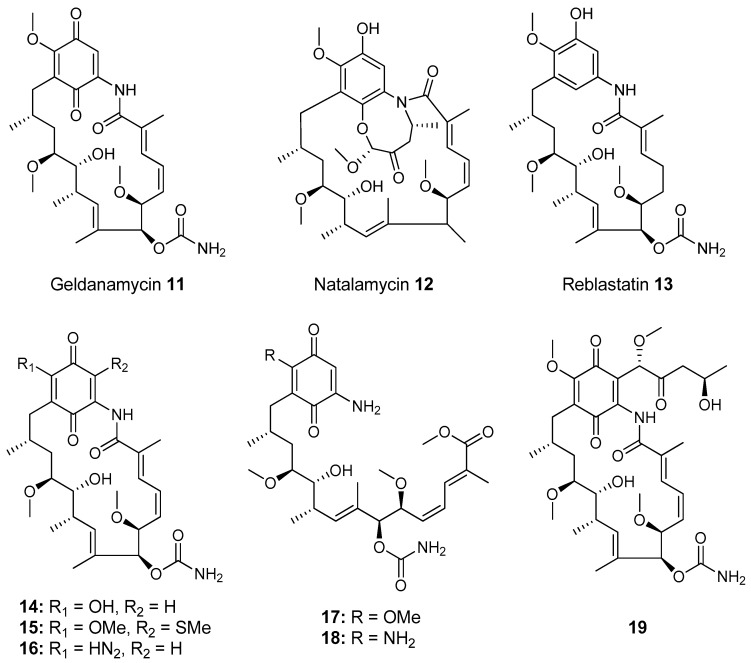
The known antibiotic geldanamycin and the related analogs discovered from a termite associate *Streptomyces*.

The structure and spectroscopic data of microtermolide A (**7**) was similar to that of vinylamycin (**9**, Figure 8), isolated in 1999 from a soil derived Streptomyces strain and prompted the structural reassignment of vinylamicin to the correct structure shown (**10**) [156] Microtermolide B **8** is a related acyclic nor derivative of **7** and is one of the only known linear depsipeptides produced by a Streptomycetes. [157] A second Streptomyces isolate M56, which is closely related to *Streptomyces malaysiensis* 1160, was isolated from the fungal comb of a South African termites nest by the same research group. This strain produced a new and highly unusual geldanamycin (**11**) analog, possessing an unprecedented [6.4.0]-bicyclic core, named natalamycin (**12**, Figure 9), as well as a number of related analogs **13**–**19** [158].

While the bulk of polyketide producing strains from termite colonies that are reported in the literature are Actinomycetes, there are some examples of non-actinobacterial producers. For example, two *Bacillus subtilis* strains were isolated from a termite colony in South Africa produced the known compound bacillaene A [159].

**Figure 10:**
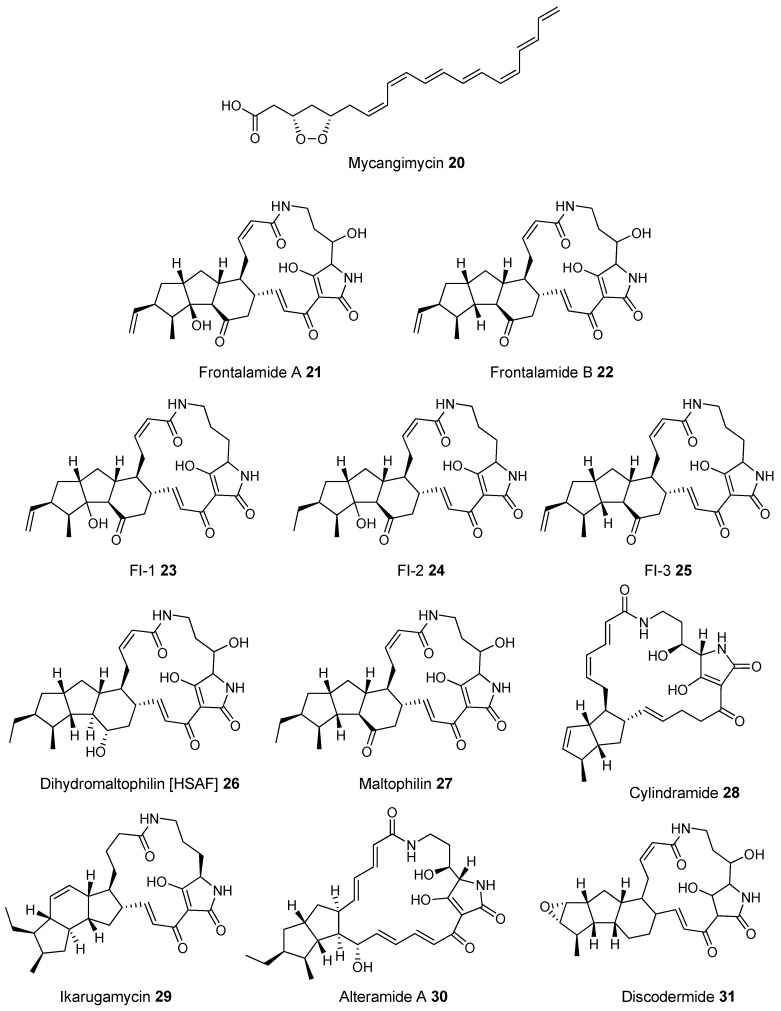
Polyketides discovered from beetle associated bacteria and known related compounds.

A third group of fungus cultivating insects is the Southern pine bark beetle, *Dendroctonus frontalis*. This beetle, a major pest of pine trees in the South Eastern United States, cultivates the fungus *Entomocorticium* for its larvae in the pine phloem. This fungus in turn suffers from the parasite *Ophiostoma minus*. Two closely related strains of *Streptomycetes* were isolated from the beetles. One was a red colony, SPB74, the other a white colony, SPB78. The SPB74 strain produced the novel polyene mycangimycin (**20**, Figure 10) which was twenty times more active against the pathogenic *Ophiostoma minus* than the farmed *Entomocorticium* [160].

After testing different cultivation conditions with Streptomyces SPB78, this strain was also shown to produce antifungal compounds. The frontalamides **21**–**25**, are mixed polyketide-non-ribosomal peptides containing a tetramic acid group and are active against *Ophiostoma minus*. The frontalamides are related to a number of known natural products **26**–**31**, including dihydromaltophilin (**26**) whose biosynthetic pathways has been previously identified the genome of *Lysobacter enzymogenes* [161]. Primers were designed based on the dihydromaltophilin gene cluster and it was confirmed that both SPB74 and SPB78 possessed similar sequences. Ultimately LCMS analysis provided support for production of a frontalamide like compound from SPB74. Dihydromaltophilin gene cluster primers also gave products from soil isolates [162].

Social insects that do not cultivate fungi may also have polyketide producing bacteria in their nests. *Coptotermes formosanus*, the Formosan termite is originally from Southern China. It is an invasive pest that has now spread to Taiwan, Japan, Hawaii and much of the South Eastern United States. It eats wood and builds large underground nests with foraging galleries that can be over 100 m long. The centres of nests are reinforced with a mixture of chewed wood and faeces known as “carton material”. Streptomyces with antagonistic activity towards termite pathogens were isolated from this carton material. These Streptomyces appeared to reduce the death rate of *Coptotermes formosanus* termites when they were exposed to fungal (*Metarhizium anisopliae*) pathogens [163]. Similarly, Actinomycetes with antagonistic activity against *Pseudomonas aeruginosa*, *Escherichia coli*, *Staphylococcus aureus*, *Serratia marcescens*, and *B. subtilis* were isolated from the nest of the paper wasp *Polistes dominulus*, an invasive European wasp [164]. However no compound responsible for these activities has been isolated.

##### Non-Social Insects as Sources for Polyketides

**Figure 11:**
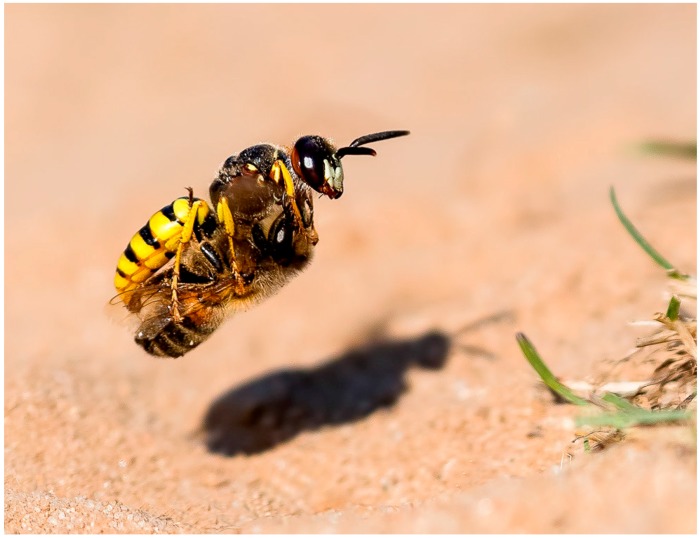
Beewolf with honey bee prey. Courtesy of Simon Jenkins http://www.simon-jenkins.photography.

Polyketide producing Actinomycetes have also been found associated with non-social insects. The larvae of some wasps are carnivorous and are laid in the bodies of paralyzed insects. In the case of the European Beewolf wasp (Figure 11), each larva is left in an underground brood cell with one to six paralyzed honeybees. Fungal infection from the surrounding soil is a frequent problem [165]. A white biomass produced in the antennal glands of the female beewolf is secreted into the brood cells which contains *Streptomyces philanthi* spores [166]. When the spores were removed many of the larvae died [167,168].

*S. philanthi* can either be vertically transmitted or acquired from the environment. *S. philanthi* strains cultured from different genera of beewolves from Europe, Asia, Africa and South America, are not resistant to antibiotics, cannot take up inorganic nitrogen, and are associated with a particular beewolf species. Because of this, they are likely vertically transmitted. In contrast the *S. philanthi* cultivated from North American beewolves are resistant to a broader range of antibiotics and share the antenna glands of their beewolves with other Actinomycetes. Because of this, they appear to have been acquired from the environment [169].

**Figure 12:**
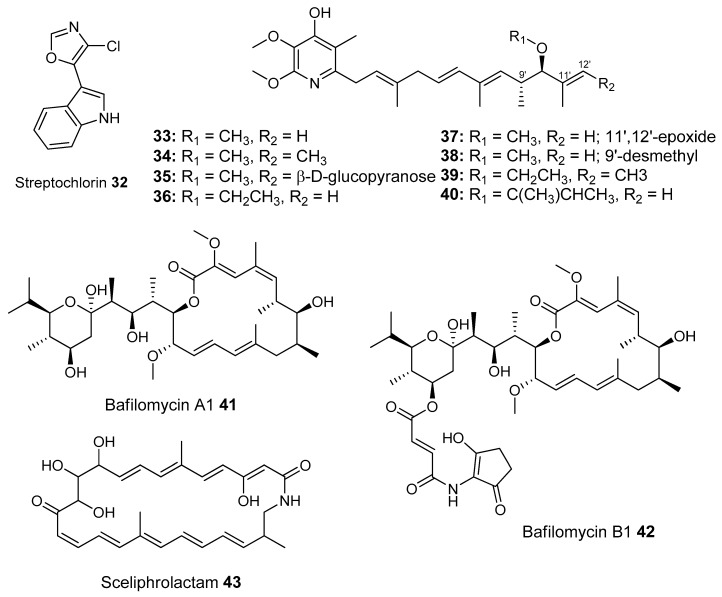
Bacterial secondary metabolites associated with non-social wasp species.

**Figure 13:**
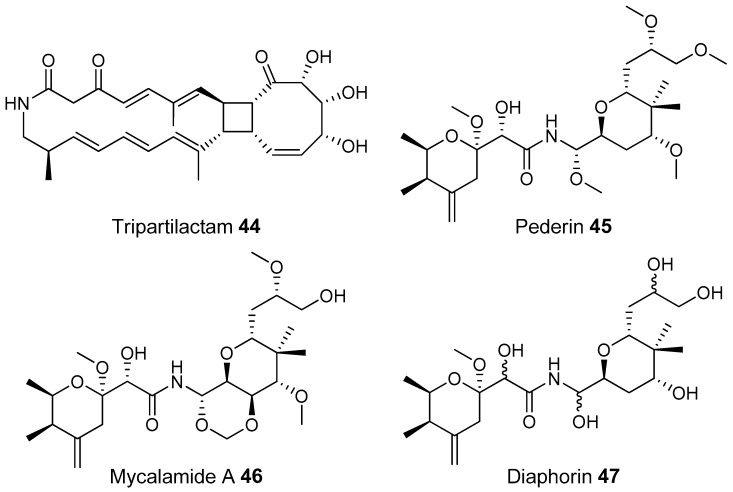
Tripartilactam and pederin from non-social beetles and related natural products from non-insect associated microbes.

Beewolf larvae are protected by several potent antibiotics, shown in Figure 12. Extracts of European *Philanthus* beewolf larval cocoons were analyzed and a cocktail of eight known antibiotics were detected. This included streptochlorin (**32**, Figure 13) and seven variants of piericidin **33**–**40**. Streptochlorin was previously isolated from the culture broth of a marine Streptomyces [170] and piericidin from the culture broth of *Streptomyces mobaraensis* [171]. This combination of eight compounds was antagonistic against a broad range of fungi and bacteria [172].

Mud dauber wasps are solitary predatory wasps that build nests from mud. Actinomycetes were isolated from two species of mud dauber wasp, *Sceliphron caementarium* and *Chalybion californicum*, and tested for production of secondary metabolites. LC–MS analyses of culture medium extracts showed the presence of a number of active compounds. While a number of these were known compounds, including the highly toxic vacuolar type H+ ATPase inhibitors bafilomycins A1 and A2 (**41** and **42**, respectively), and mycangimycin (**20)**, which was also found in Southern Pine Bark beetles [173], a new polyene macrolactam, sceliphrolactam (**43**), was also discovered. This compound has potent antifungal properties against amphotericin B-resistant *Candida albicans* [174].

The Dung beetle lays it’s larvae in a pellet of faeces, another environment with potential for infection. A Streptomyces (*Streptomyces* strain, SNA112) from a pellet used by *Copris tripartitus*, the Korean dung beetle, produces tripartilactam (**44**, Figure 13), a novel tricyclic lactam. The compound is inactive against all bacteria, fungi and cancer cells that it was tested against, but does inhibit Na^+^/K^+^ ATPase [175].

The best known case of a bacteria living on a non-social insect producing a polyketide (in this case a mixed NRP type I PKS) is the case of pederin (**45**) found on Paederus and Paederidus beetles. Pederin causes dermatitis on the human skin and appears to deter predators from eating the beetles, and has strong anti-tumour activity. Pederin is produced by a *Pseudomonas* (a gamma proteobacteria) that is transferred vertically through female beetles. Both Pederin itself and its intermediates are similar to mycalamide A (**46**) made by a bacteria that inhabits marine sponges [176]. Recently a compound highly similar to pederin (diaphorin (**47**)) was found to be produced by a β-proteobacteria which grew on the Asian citrus psyllid *Diaphorina citri* [177].

Insect-associated bacteria are the most studied of the environments that we review in this article. A large literature is available to help pick where to sample. The main question in picking samples is whether the kind of insect or the environment that the insect produces is more important. As some of the social insects discussed here are pests (attine ants, Southern Pine Bark beetles, the Formosan termite), plant protection or pest control offices may be able to help find sampling sites.

#### 3.1.2. Desert Soils

Deserts are areas with less than 200 mm of precipitation a year and they can be found from the equator to the poles. Deserts may have arid or hyper arid climates. An arid climate has a ratio of mean annual rainfall (MAR) to mean annual evaporation (MAE) of 0.2–0.03 and precipitation of 100–300 mm/year. In hyper arid areas, where there is no vegetation, the MAR/MAE ratio is less than 0.03 and there is less than 100 mm of precipitation/year [178].

Several research groups are bioprospecting desert soils for pharmaceutically useful secondary metabolites as desert soils are often enriched in Actinobacteria. Actinobacteria have been found to be the most common group in an Antarctic desert soil [179] and Atacama desert soils [180,181] and the second most common group in a Saharan soil [182]. This is not the case for all studies though, their relative percentage in the bacterial community has been found to vary with season in the Negev [183]. Furthermore in a study of soils of the Gobi and Taklamakan, the Actinobacteria were found to be relatively unimportant compared to the Bacilli [184].

The most reliable assessment of desert soils for actinobacterial enrichment and the presence of polyketide synthase genes are studies that compare a range of desert soils with other soils using molecular methods. This minimizes bias due to primers and sample processing. Fierer et al. in 2012 [72] compared soil DNA from three hot arid deserts from the Southwestern United States with soil DNA from six Antarctic cold hyper-arid deserts and seven non desert soils (arctic tundra (1), prairie (1) and tropical (2), temperate (2) and boreal forest (1)). DNA was characterized by next generation sequencing of 16S amplicons and shotgun metagenomics sequencing. According to both metagenomic shotgun sequencing and amplicon sequencing, the percentage of Actinobacteria was highest in cold desert soils, followed by hot desert soils. Fierer et al. ascribed this to the high pH and dryness of these environments. The bacterial communities of the non-desert soils were relatively similar to each other. The bacterial communities of cold and hot desert soils differed both from each other and non-desert soils. 16S sequence results found that hot deserts had comparable diversity to other non-desert soils, while hyper arid Antarctic cold deserts were less diverse.

Xu et al. 2014 [185] reanalyzed Fierer et al.’s [72] results with literature data from 17 other sites, including 12 English grassland and a Brazilian Mangrove soil. Again this study showed that desert soils were enriched in Actinobacteria, although most were not *Streptomyces*, but rather *Bifidobacteriaceae*, *Mycobacteriaceae*, and *Frankiaceae*.

One could argue the relatively large actinobacterial fraction of the bacterial community means that these soils are a good source of new polyketide natural products. In contrast, Fierer et al. [72] found that fewer of the metagenome shotgun sequences in his study were implicated in virulence and defence, including antibiotic resistance, compared to other soils. They suggested that the harsh desert conditions are less conducive to bacterial growth, reducing microbe-microbe competition, leading to less antibiotic production and resistance.

The Brady laboratory used environmental DNA from hot desert soils from the same area to bioprospect for natural produces and came to the opposite conclusion. Powers et al. [105] compared soil DNA from desert, forest, farmland, grassland and salt-water marsh soils by amplifying with 16S, NRP A domain, and PKSII KSα specific primers. Sampling sites were in the US Southwest and New England. The KSα diversity, (from type II aromatic polyketide biosynthesis) correlated well with the 16S actinobacterial fraction of the soil bacterial community. Most known PKSII producers are Actinobacteria. The study found that arid soils contained the most diverse KSα domains and that New England forest and salt march soils contained the least.

**Figure 14:**
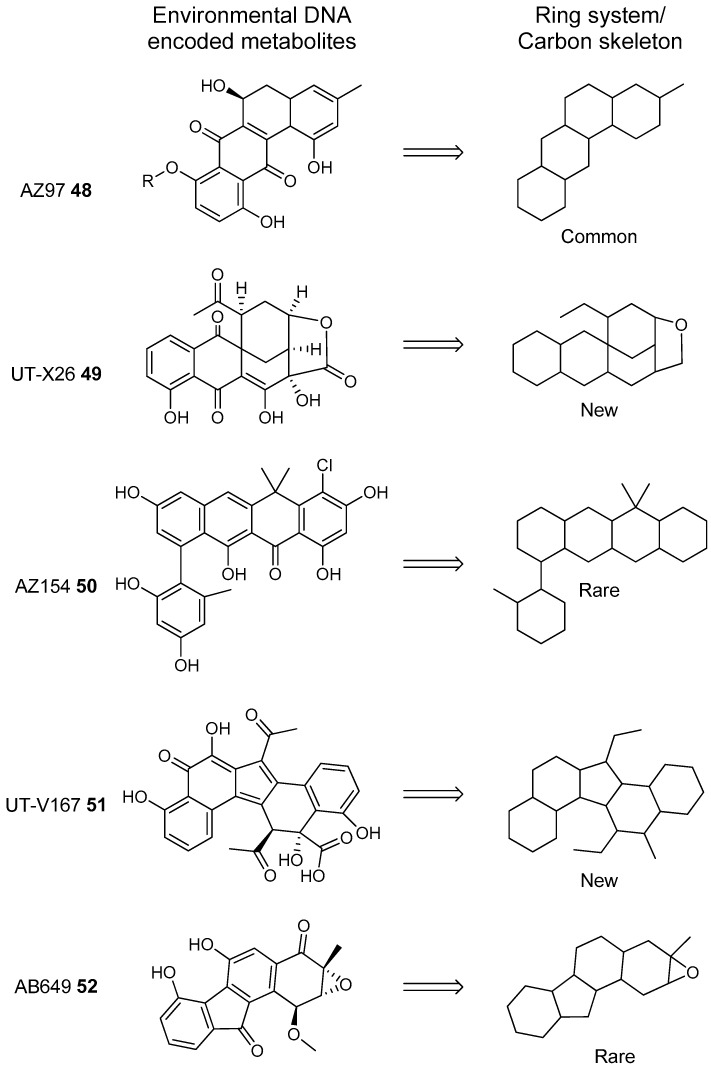
Type II polyketides found in metagenomic libraries from desert soils and their ring systems. Reproduced with permission from Feng et al., *Proc. Natl. Acad. Sci. USA.*, published by United States National Academy of Sciences, 2011 [187].

The environmental DNA from these desert soils were then used to make metagenomic libraries that were screened with primers for the KS domains of type I [186] and type II [187] Polyketide synthase pathways. This work discovered of several novel aromatic type II polyketides. Three environmental clone libraries from California [188], Arizona, and Utah [187] were screened with Type II specific primers and five Type II pathways were identified and heterologously expressed in Streptomyces. One of the pathways produced a compound similar to the known polyketide landomycin E, an angucycline (**48**, Figure 14) [188]. The four remaining pathways produced far more unique compounds **49**–**52**. Two had new ring systems, a novel naphaquinone called erdacin (**51**) and a new aureolic acid (**49**). The structures of the last two compound were rare variants of the angucyclines (**52**) and pentangular polyphenol **50** [187].

**Figure 15:**
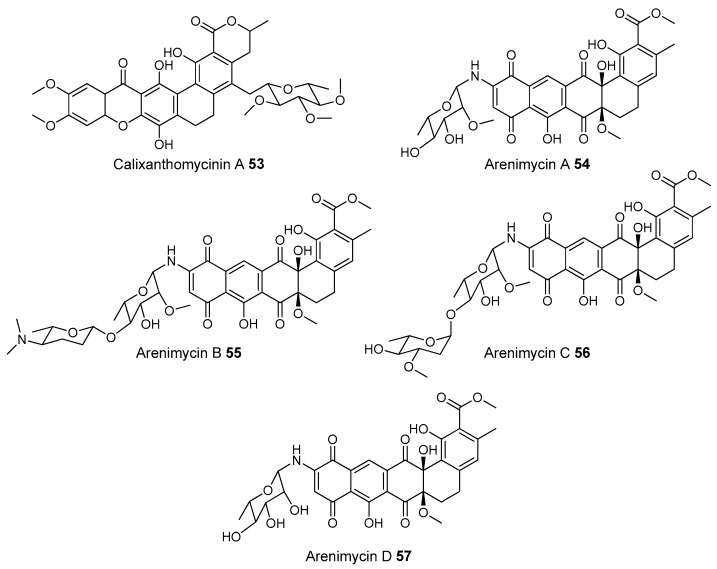
Pentangular polyphenols discovered from desert soil derived metagenomic libraries.

Since this [187], the Brady group has screened desert soil metagenomics libraries for more pentangular polyphenols [19,189] and have found arixanthomycins, calixanthromycin A (**53**, Figure 15), and arenimycins C (**56**) and D (**57**). Desert soils are promising for aromatic type II polyketide discovery.

**Figure 16:**
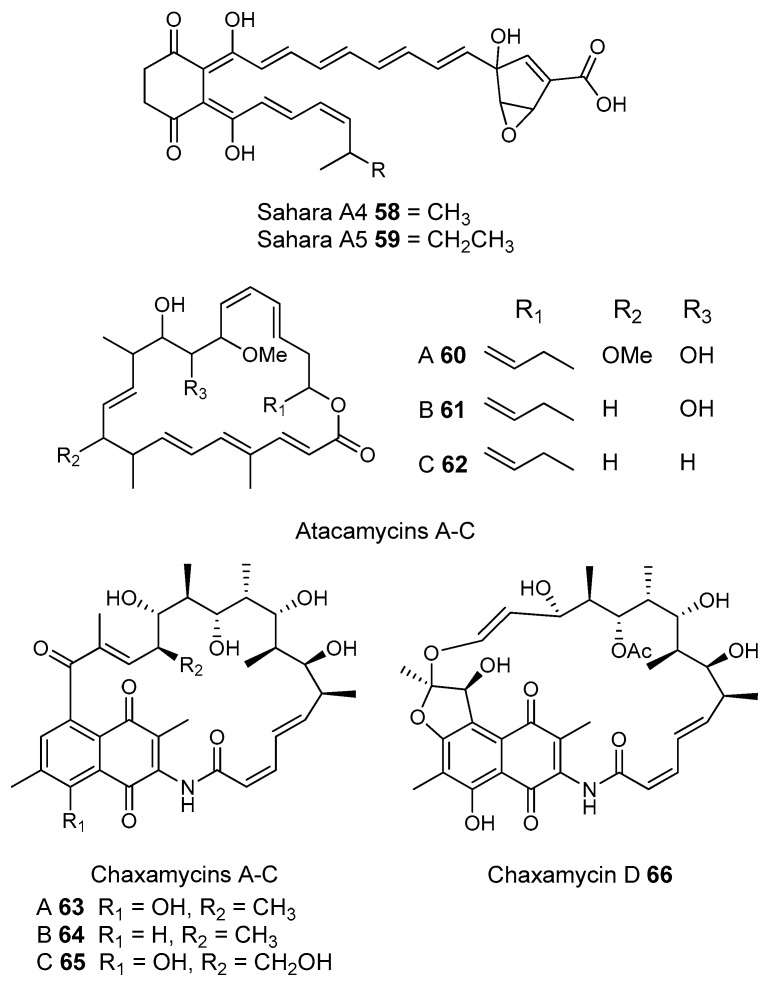
Natural products from desert isolates.

Several other groups have found polyketides in desert soils by culturing Actinomyces from them. Several non-streptomycetal actinomycetal isolates from the Algerian Sahara have been found to produce antibiotics. An *Actinomadura* isolate was cultured and made an antifungal agent, however the final structure of the compound was not determined [190]. Similarly a *Streptosporangium* isolate produced several of presumed glycosylated aromatics natural products with antibacterial activity against Gram-positive bacteria [191]. Again the structure of these compounds was not fully determined. Lastly a *Saccharothrix* isolate also from the Algerian Sahara made two similar highly novel polyketides (**58** and **59**, Figure 16) with potent antifungal effect on some filamentous fungi but not yeasts [192].

The Atacama Desert in Northern Chile is the driest and oldest non-polar desert in the world. Most of the Atacama desert is hyper-arid and the most significant source of water is sea fog that condenses and supports limited plant life. There is an extremely hyper arid valley near the Yungay region where the MAR/MAE is less than 0.002. Fog does not reach this area because of a mountain barrier between it and the Pacific Ocean. This region has no vegetation and soils have very little organic matter. It is often impossible to detect bacteria in these soils which are often described as Mars like [193].

Five studies have used molecular methods to characterize the bacterial community phylogeny of Atacama Desert soils and found that the bacterial communities of these soils are very different from other desert soils. When Fierer et al. compared the bacterial communities of deserts and other biomes they found that most hot desert soils were as or more diverse than soils from forests and grass lands (8000–12,000 OTUs). Hyper-arid Antarctic soils could have half this number of OTUs [72]. Soils from the Atacama Desert are orders of magnitude less diverse than this. Drees et al. [194] compared the bacterial community of soils in the core arid region with more humid areas closer to the ocean and further inland at higher altitudes using a 16S community fingerprinting method. There were two distinct clusters of fingerprints, one from the core arid region and a second from a coastal sample and samples from higher up in the mountains. This same research group from the University of Arizona, later used next generation sequencing of 16S amplicons to characterize three samples taken from the edge of the Mars-like core of extreme hyper-aridity. In these samples 70 and 77% of sequences were actinomycetal. However, the community structure was far less diverse than that of most soils in Fierer’s study with 123 and 142 OTUs found in 2200 and 3560 sequences, respectively [180].

Two studies using cloning and sequencing of 16S amplicons have found that the bacterial community of soils from the hyper arid core were dominated by a single clone. In one case the community was almost entirely actinomycetal, with sequences ranging from 91% to 95% similarity to a known Frankia genus making up 94% of sequences [181]. A second study [195] however only found bacilli in 244 clones from a single sample. 154 of these 244 amplicons were a single sequence.

A recent report describes three even more arid areas in the Atacama Desert than the Yungay region. The authors report that the bacterial community as determined by 16S community fingerprinting as being actinobacterial with sequences from Actinobacterium, Aciditerrimonas and Geodermatophilus genera, Proteobacteria, Firmicutes and Acidobacteria [196].

In November of 2004 Bull, from the University of Kent, sampled three sites in the Atacama desert in arid, hyper-arid, and extremely hyper arid regions [197]. These samples were used to culture Actinobacteria using selective media and 46 strains were found from the *Amycolatopsis, Lechevaliera* and *Streptomyces* genera. Two Streptomyces strains from the hyper arid site (C34, C38), a salt flat known as the Salar de Atacama were closely related to *Streptomyces leeuwenhoekii*. Each of these strains has been a source of a novel type I polyketide family, the atacamycins and the chaxamyins.

The atacamycins are a family of 22 membered macrolactones [198] produced by strain C38. There are three closely related forms of atacamycin A, B and C (**60**–**62**, respectively, Figure 16) that vary by oxidation of a common macrolactone ring. These compounds were weakly inhibitory to *Ralstonia solanacearum* but atacamycin A and B showed cytotoxic activity against several human tumour cell lines. Strain C34 produced four members of the chaxamycin family, compounds A–D (**63**–**66**). Chaxamycins are napthalene ansamycins. Chaxamycin D (**66**) showed activity against of methicillin-sensitive and resistant *S. aureus* (MRSA) although it was not as active as rifampicin. Chaxamycins A–C were inhibitory toward ATPase activity of human Hsp90, a test of anti-tumour activity for ansamycins [199].

Both metagenomics and culturing are thus finding new actinobacterial polyketides from desert soils. The soils of the hyper arid Atacama Desert have a distinct bacterial community to that of deserts such as the Sahara or the Mohave and it is not yet certain how this affects the polyketides that their bacterial communities produce.

#### 3.1.3. Disease Suppressive Sediments

Soils that suppress disease have been a target for antibiotic polyketide discovery. In the most direct sense disease suppression can mean using soil to cure human disease, such as red soils from the Mediterranean climate region of north western Jordan. These soils are used to treat skin infections in traditional medicine. When inoculated with *Micrococcus luteus* and *Staphylococcus aureus*, bacterial isolates from these soils are more likely to produce antibacterial compounds including actinomycin [200]. Usually though disease suppressive soils refer to soils on which soil borne plant diseases, particularly fungal pathogens, are less likely to infect crops.

Disease suppression alone does not mean that a soil is a good target for bioprospecting. Suppression can be caused by a soil property such as pH. Certain crops can also produce chemicals which suppress disease. Even when suppression is caused by the soil microbial community this does not necessarily mean that antibiotics are being produced. Any soil that is sterilized will become much easier for a pathogen to colonize as it does not have to compete with microbes already there. The suppressivity of the non-sterile soil is called natural suppression and is caused by the whole soil microbial community [201]. In contrast induced suppression occurs when certain crops are grown, certain crop growing sequences are used, or the crops are managed a certain way [202]. Induced suppression can be due to antibiotic production.

The different roles of natural and induced suppression are reviewed by Janvier et al. [203]. The test of microbial induced suppression is if sub-samples of this soil can be mixed with non-suppressive soils making it suppressive to a particular pathogen and if the suppressive effect of a soil is destroyed by autoclaving. If this is the case, the microbial community of these soils is probably causing induced suppression.

Two publications suggest suppressive soils as a good target for antibiotic bioprospecting [204,205] but recommend very different kinds of soil. The European Union funded METACONTROL project bioprospected disease suppressive soils to find new antibiotics. Five suppressive soils from across west Europe were used. The most suppressive of them was a grassland that had recently been converted to arable land [206]. Researchers ascribed this suppressiveness to the biodiversity of the soil plant cover and microbes [207]. In contrast other studies have found that soil suppressiveness increases under monoculture, when a single crop is grown at the same site for a long time [202,208]. These soils were also suggested as a target for bioprospecting for antibiotics [205].

The suppressive effect of these soils under long term monoculture has been ascribed to the effect of coevolution [209]. If a range of plants are grown on a soil, there are many different kinds of food for the microbial community. Ecological niches can develop as certain microbes specialize in certain substrates. If a single crop is grown over a long period there is a much narrower range of substrates for microorganisms and there is more competition and inducement to use antibiotics. As these conditions persist, there is an “arms race” between bacteria as they use more of their genome to produce more diverse antibiotics.

Kinkel et al. [209] list other factors that encourage the development of soil suppressivity. The soil should receive a lot of readily available substrate for the microbial community to consume. Readily available substrates ensure that the microbial community can produce the metabolically expensive secondary metabolites. Additionally readily available substrate ensures high population densities, encouraging antagonism. Finally moderate ploughing will distribute antibiotic producing bacteria through the soil where they can outcompete other microorganisms that specialize in particular soil niches. However, too much ploughing can homogenize soil microbial communities to the point where there is less coevolution and thus fewer new antibiotics.

Soils can be made suppressive by adding organic matter to the soil such as manures, green manures (plants that are grown and then ploughed into the soil), peat, and compost [210,211]. The best known and most effective of these organic materials is compost [212]. The suppressive effects of compost in soil vary depending on the stage of compost development and the materials used [213,214]. Antibiotic production is thought to be one several ways that compost microorganisms suppress disease, with the others including competition for nutrients and parasitism [212].

Hadar and Papadopoulou [212] review coevolution and suggest that this probably does not explain the suppresive effect of compost because the bacteria in compost are not in the soil long enough to coevolve with the plants and microbes in the soil. Instead they suggest that as raw organic matter develops into compost certain microbial groups are selected for. The actinobacterial fraction of the soil bacterial can increase after the adding manure [215], although others recommend the addition of organic amendments as they provide the conditions for suppressive bacteria to grow [216]. A review of over 2000 studies of the suppressiveness of organic amendments found that that while actinobacterial prevalence had an effect on suppressivness, it had less of an effect that total bacterial and fungal prevalence [211] suggesting that some of the suppresive effects of compost microbes is due to natural suppression.

There is evidence that composts can produce antibiotics. Sometimes instead of adding compost directly, it is soaked in water for several hours or days and the supernatant, called compost tea is sprayed on the plant leaves to prevent plant disease (see [217] for full discussion). This compost tea can retain much of its antifungal effect after pasturization [218] or passage through 0.1 or 0.22 µM filtres [219,220], suggesting that it is due to small molecules. The authors were careful to point out that their result was caused by the growth of anaerobic microbes [219] and that other compost teas made with different ingredients were rendered ineffective by filtration and/or sterilization [194,219]. Thus, these result may be due to induced systemic resistance rather than antibiotic production [218].

Composts go through several phases as they mature. There is an initial hot phase (thermophilic), after which the compost cools (mesophilic) and matures. It is generally thought the actinobacterial fraction of the bacterial community increases during the later stages of compost development [213,214,221]. A recent study that used next generation sequencing of 16S amplicons to characterize the bacterial community of three forms of compost, found that the actinobacterial community fraction decreased during the thermophilic phase and increased during the mesophilic phase [43]. Fatty acid–based community profiling has also shown that the actinobacterial community changes composition as compost develops [187]. Compost may be a good target for polyketide bioprospecting but its bacterial community is continually changing.

**Figure 17:**
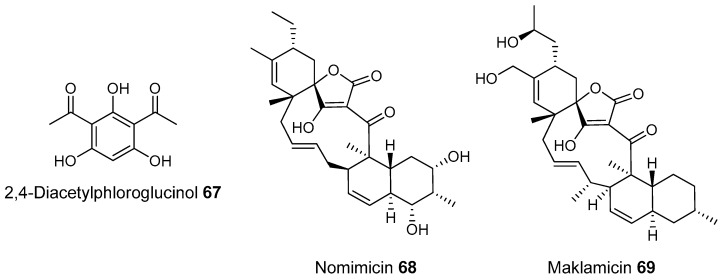
Natural products from disease suppressive soils.

Several studies have identified antibiotics, including polyketides, produced by bacterial isolates from compost. A strain of *Pseudomonas aeruginosa* supressed a fungal pathogen *Fusarium oxysporum* infection of cucumber [222]. Suppression was due to 2,4-diacetylphloroglucinol (**67**, Figure 17), a previously known type III polyketide [223]. Pseudomonads are important antibiotic producers in both suppressive soils and compost [221,224].

As well as the spirotetronate type I polyketide, nomimicin (**68**) was discovered from a non-streptomycetal Actinomycete (*Actinomadura*) that was isolated from compost [225]. Nomimicin is similar to the already known maklamicin (**69**). The genomes of two Streptomyces isolated from compost are also being sequenced. Both contain a number of interesting polyketide synthase pathways suggesting these strains may produce new compounds [226,227].

Little polyketide bioprospecting has been done in disease suppressive environments compared to others listed here. Disease suppressive sediments include a broad range of environments, natural soils, intensively cultivated soils, and composts. They can thus have many different bacterial communities. Determining if a sediment is disease suppressive and whether this due to bacterial production of antibiotics requires either greenhouse testing and/or knowledge of plant disease history on a particular piece of land. Bioprospectors will need to work with agriculturalists to identify suppressive soils and sediments.

#### 3.1.4. Caves

There are several reasons for bioprospecting for polyketides in caves. Caves represent isolated and stable environments where bacteria can evolve independently of life on the surface [228], and also develop long term chemical “arms races” between each other [205]. For example, bat guano can build up over hundreds of years [229] providing a stable and nutrient rich environment in which insects and streptomycetes can flourish [230]. However, most caves are nutrient poor (oligotrophic) [231] and this lack of nutrients may encourage cooperation and interaction between microbes in caves rather than competition [232].

Usually the proteobacteria are the largest phylum in cave bacterial communities, but cave isolates are often actinobacterial [233]. This is not always the case though, some cave bacterial communities are highly actinobacterial [234,235] and the majority of these Actinomycetes are often Pseudonocardia. Tomczyk-Zak and Zielenkiewicz [236] reviewed the distribution of different bacterial phyla in caves. Communities dominated by Actinobacteria tend to be found on cave walls and in crystal structures such as stalactites and stalagmites. Between 2000 and 2009, 34 new species of Actinomycetes were isolated from caves, including several new genera [233]. Caves may contain many novel actinomycetes that may also produce novel polyketides [237].

Several different processes can lead to cave formation. Most caves are found in karst landscapes that cover around a fifth of the worlds land area [231]. Karst landscapes develop when slightly acidic rainwater gradually dissolves carbonate rock such as limestone. Two novel aromatic type II polyketides have been characterized from Actinomycetes isolated from karst caves.

**Figure 18:**
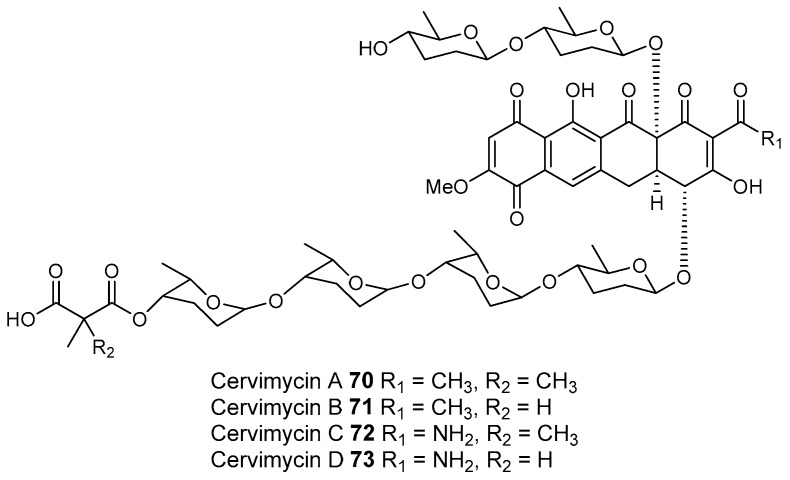

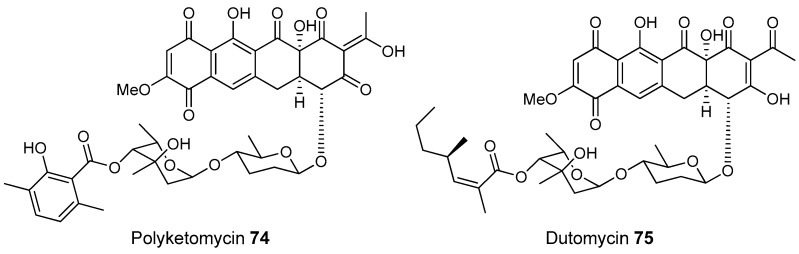
Cervimycins, isolated from cave derived Actinomycetes, are related to known tetracycline antibiotics **74** and **75**.

The Groto de Cervi is an organic matter-rich cave in Southern Italy with extensive bat guano deposits. Bacterial isolates from throughout the cave are often Actinomycetal. The cave also has 5000 year old Neolithic paintings in ochre and guano [238]. An isolate similar to *Streptomyces rochei* was isolated from a guano painting. This isolate was found to produce four type II polyketides (Cervimycins A–D, **70**–**73**, Figure 18) with activity against multidrug resistant *Staphylococcus aureus* and vancomycin resistant *Enterococcus faecalis*. All of the cervimycins contained a central four ring structure similar to the tetracyclines that is bis-glycosylated [239,240].

**Figure 19:**
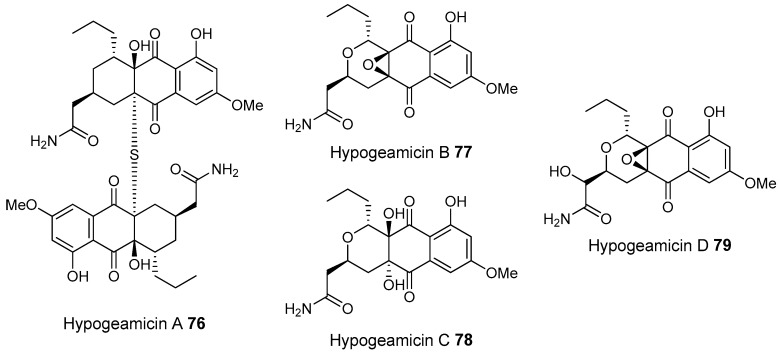
Type II polyketides isolated from an Actinomycetes isolate from Hardin’s cave.

Hardin’s cave is a relatively small organic matter rich karst cave in Tennessee, also with a large bat population. An Actinomycete, *Nonomuraea specus*, was isolated from a piece of decomposed bark in the cave. *Nonomuraea specus* produces a sulphur-bridged dimeric pyronaphthoquinone, called hypogeamicin A (**76**, Figure 19). The non-dimeric precursors **77**–**79** were also isolated and were weakly toxic to *Bacillus subtillis*. The dimeric product **76** is toxic to TCT-1 colon cancer cell line. This is similar to the cytotoxicity of the related sulphur bridged dimer BE-52440 series (half maximal inhibitory concentration IC_50_ = 6.4–12.8 μM) but significantly lower than that of paclitaxel [241].

**Figure 20:**
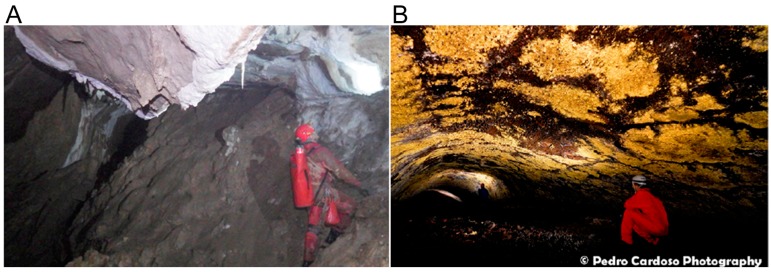
(**A**) An example of Moonmilk (above) in Goatherds Chasm in Switzerland. Photograph provided by Olivier Gallois of the Groupe Spéléologique Archéologique Mandeure; (**B**) Yellow Microbial mats from the volcanic cave Gruta de Terra Mole in the Azores. Photo courtesy of Pedro Cardoso. Reproduced from Riquelme et al., *Front. Microbiol.*, 2015 [248].

“Moon milk” is often found in karst caves (AKA mondmilch, Figure 20A). Moon milk is one of several kinds of cave deposits that is formed by the dissolution and reprecipitation of carbonates. Bacteria are thought to be involved in moon milk precipitation. Moon milk is made of calcium or magnesium carbonate and its texture can range from paste to powder [242]. There is a long history of moon milk use in medicine that dates from at least 1555, which led to it being exhaustively mined from some European caves [243]. A Russian expedition to a large karst cave formation in Siberia isolated *Streptomyces* and *Nocardia* from cave moonmilk that produced of antibacterial and antifungal compounds. One of these compounds chaxamycin B (**64**), was previously found in the Atacama desert (discussed earlier in this review, Section 3.1.2) [244].

A rarer and shallower form of caves are lava tubes, which form in volcanic areas when the surface of lava solidifies and the underlying molten lava continues to flow [231]. These caves are usually in basalt and can receive organic material from the surface through tree roots. Several researchers have bioprospected for antibiotic producing Actinomycetes in volcanic lava tubes in the Azores [245] and British Columbia, Canada [246] and found isolates with antibacterial activity. Coloured microbial mats are often found on the surface of volcanic cave walls (Figure 20B). 16S amplicons from DNA of coloured microbial mats from volcanic caves in the Azores and Hawaii were sequenced and the data showed that Actinobacteria were one of the major phyla present [247]. A comparison of the actinobacterial fraction from volcanic caves from the Azores, Hawaii and New Mexico found that most (74%) of the 16S sequences were from five OTUs, with the two most common 16S OTUs (59%) being from *Pseudonorcardiaceae*. However most OTUs (71%) were a single sequence. The authors interpret this to indicate that much of the actinobacterial richness in caves is derived from species unique to those particular environments [248], suggesting that sampling of many different caves is likely to yield new Actinobacteria and new polyketide natural products.

#### 3.1.5. Extremophiles

The main argument for looking for bacterial polyketides in environments with extremes of pH, salinity and heat is that while soil has been sampled to the point of rediscovery, extreme environments are poorly studied. Differences in the environment will mean that there are differences in the secondary metabolites produces by the bacteria found in these environments [249].

**Figure 21:**
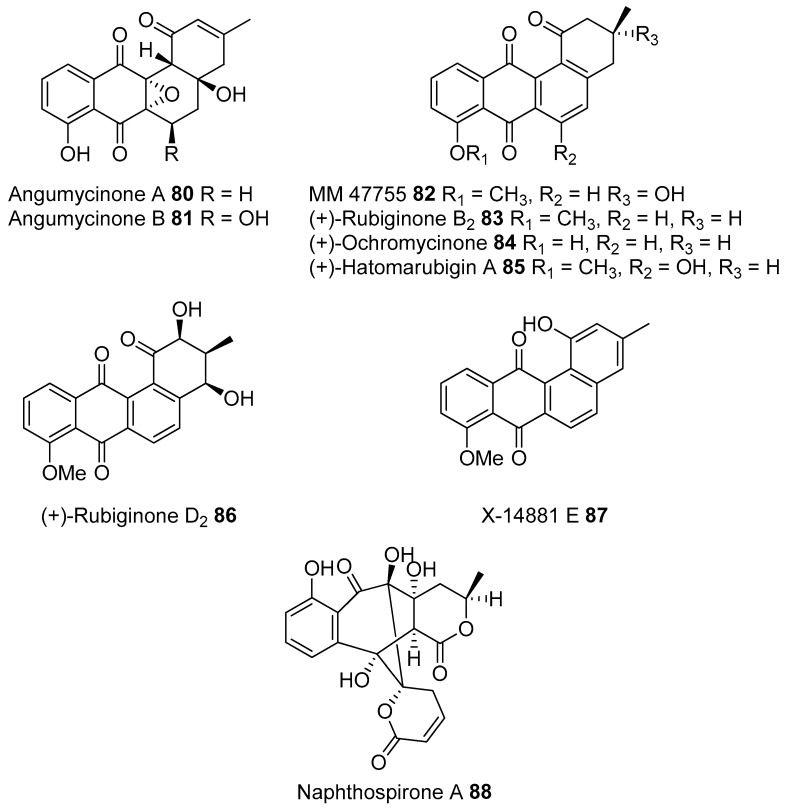
Polyketides discovered from cultured Actinomycete strains isolated from highly acidic mine drainage and highly basic mine tailings.

Extreme pH environments can often be found in abandoned mines, such as an abandoned coal mine in South Korea where sulphides are oxidized on contact with the atmosphere. Here the mine drainage is at pH = 3. A Streptomycete isolate from this acid mine drainage produced eight type II aromatic polyketide angucyclinones (**80**–**87**, Figure 21) [250]. These compounds were tested against several bacteria and found to have antimicrobial activity against the Actinomycete *Micrococcus luteus* and Firmicutes *Enterococcus hirae* and MRSA.

Mines can also be highly alkaline. An Actinomycete from the genus *Nocardiopsis* was isolated from a tin mine tailings in southern China with a pH of 10 and was shown to produce the structurally unprecedented compound naphthospironone A (**88**). This highly unusual spiro[bicyclo[3.2.1]octene-pyran]dione ring containing compound was moderately active against a small panel of cancer cell lines, as well as several Gram positive and negative bacteria [251].

**Figure 22:**
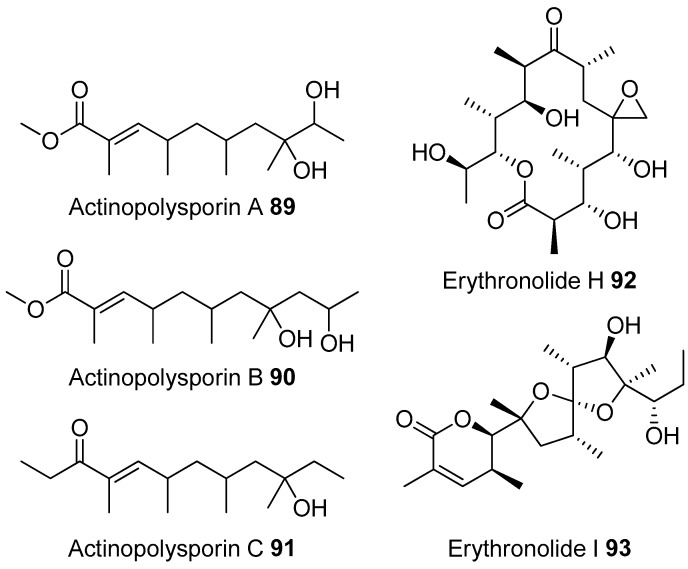
Polyketides identified from *Actinopolyspora erythraea*.

Jose and Jebakumar briefly review bioprospecting the Actinomycetes of hypersaline environments [249]. Two new species *Actinopolyspora alba* sp. nov. and *Actinopolyspora erythraea* sp. nov. were isolated from the Baicheng salt field in Xinjiang province, China [252]. *Actinopolyspora erythraea* produces several interesting polyketides. Actinopolysporins A–C (**90** and **91**, Figure 22) are novel polyketides; unfortunately with no detectable biological activity [253]. *Actinopolyspora erythraea* also produces two congeners of erythromycin, erythronolide H and I (**92** and **93**, respectively).

### 3.2. Potential New Environments for Bioprospecting

We have reviewed some well cited terrestrial environments that have produced new polyketides. However, this is by no means a definitive list. We will end this section by describing two environments that have been suggested for polyketide bioprospecting as they are enriched in Actinobacteria but have not yet yielded any new polyketides.

#### 3.2.1. Cities

There are several arguments for bioprospecting in cities. Soils in cities may contain a broad range of bacteria as they are continually introduced by goods and travelers and are managed in a wide range of ways (2.5). Cities may also select for Actinobacteria, particularly in environments such as street dust or stone surfaces.

Two recent studies (discussed earlier in the section on biogeography) from well-known research groups, characterized Park soils of New York City. A next generation sequencing study of 16S amplicons by the Fierer laboratory found that most of the 16S amplicons present in a broad range of soils from many climates could be found in Central Park soil [122]. A very recent next generation sequencing study of type I PKS ketosynthase domain amplicons, by the Brady laboratory, from several New York parks found that while there were park soil specific communities, synthase sequences for a range of secondary metabolites that were originally isolated from across the world could be found in New York parks. The Brady laboratory suggests, that this is not a property of city soils but of most samples and that it might be more useful to screen a few samples deeply rather than shallowly screen many [123].

While cities are usually less biologically diverse than rural areas, this is not true for all organisms at every scale. Plant communities of suburban areas can be more diverse than rural or downtown areas [254,255]. Several studies have found that some eukaryotic organisms are more diverse in cities. This has been ascribed to continuous introduction by humans as seen with earthworms in Australia [124], a broader diversity of environments as seen with Clitellate worms in Stockholm [256], and a greater range of substrates as seen with fungi colonization of a stone surface in Vienna [125].

It is also possible that cities are also enriched in Actinobacteria. The Central Park New York 16S sequencing study, found that the microbial community in the soils of Central Park was enriched with Actinobacteria compared to a broad sampling of soils from across a wide range of eukaryotic biomes [122]. Our comparison of the bacterial communities of forest, cultivated soils and street dust found using cloning and sequencing of 16S amplicons showed that street dusts were enriched in Actinobacteria [46]. When a subset of these samples was amplified with PKSI specific primers, PKSI pathways that were actinobacterial seemed to be selected for in street dust while non-actinobacterial pathways were selected for in soil [104]. Actinobacteria are known to be selected for by nitrogen, and urban environments receive more nitrogen from the atmosphere than the countryside [257], which may affect biological processes in urban soils [258]. Our laboratory is currently working on a Streptomycetal isolate from a bus stop. Its genome has 35 secondary metabolite pathways. [259].

#### 3.2.2. Airborne Bacteria

**Figure 23:**
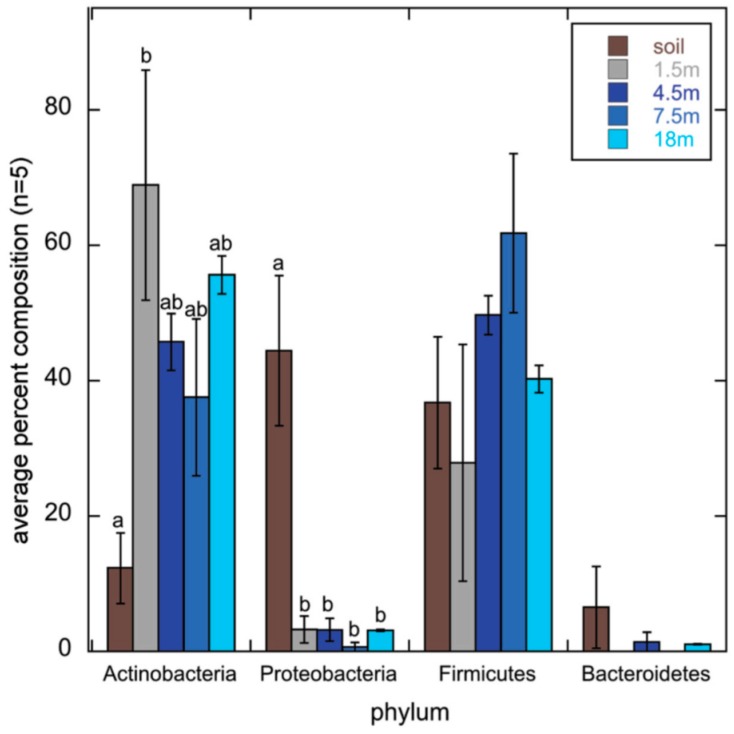
Average percent composition at the phylum-level classification of bacterial communities at the soil surface or at 1.5, 4.5, 7.5, or 18 m above the surface. Reproduced from Weber and Werth, *Front. Microbiol.*, 2015 [260].

Weber and Worth used 16S next generation sequencing to compare the bacterial communities of soils with the bacteria from the air above them (1.5–18.0 m). The sample site was in a small city in Idaho. More airborne bacteria were actinomycetal compared to the top 2 cm of soil (12% versus 38–69%) and while half of soil Actinomycetes were *Streptomycetes* most airborne Actinomycetes were from other groups (Figure 23). The authors suggest that the airborne bacterial community has many Actinomycetes as their spores are easily carried by air (spore forming Firmicutes made up most of the rest of the bacterial community) [260]. As *Streptomycetes* have already been extensively exploited for polyketide discovery [237], collecting bacteria from the air could be an simple mechanism for selecting novel non-streptomycetal Actinomycetes for bioprospecting.

## 4. Conclusions

### 4.1. Taking Samples

Finding new polyketides in the environments essential as antibiotic resistance increases. Sampling is the first step in this. For reasons of cost and convenience most polyketide bioprospecting will be on land [260]. Understanding the questions that Microbial ecology can and cannot answer about bacterial and polyketide distribution will help natural product chemists sample. The first section of this review covers these uncertainties which make it difficult to decide how to sample for polyketides in terrestrial environments.

The second section of this review lists several of the environments that have been prospected for new bacterial polyketides, either through isolation or metagenomics methods. The research groups that do this usually specialize in a single environment that they sample extensively, whether insect associated bacteria, caves, or deserts soils. This may limit discovery. We have few comparisons of bacterial populations or polyketide synthases from very different environments such as soil versus compost or cave sediment, until we do it is not wise to invest heavily in a single environment. It is still unclear if most bacterial or polyketide synthase distribution is controlled by biogeography. If they are not, then there is little value to sampling a habitat more than once.

Several studies have found the similar polyketide synthases in very different environments, such as sea sponges and insects [176] or deserts and caves [244] so it is possible that everything is everywhere in an absolute sense. Comparisons of polyketides between different soils [59,93,121] and soils/street dust/vermicompost [104] have found that at least certain polyketides are more common in certain environments.

An efficient way to access many new polyketides quickly would be to sample (isolates and/or metagenomics clones) at a low level many environments with contrasting polyketide synthases. This will mean taking many samples and choosing a subset of them that have plentiful and contrasting polyketide synthases. The first step is to pick samples well. This will often mean talking to people who know more about the environment to be sampled than the bioprospectors themselves, such as plant protectionists, cavers, pedologists, compost producers, or street sweepers. For soils, at the local level the best sources of information are soil surveys and surveyors. A good overview of how soil properties vary at higher levels is http://soilgrids.org/, which predicts soil properties such as pH at a one square kilometre resolution. Several studies have mapped the distribution of bacterial communities in soil at the regional, national or continental scale [55,56,261]. The Earth microbiome project is comparing the bacterial communities of contrasting environments [37].

### 4.2. Characterising Samples

The second step is to characterize samples. Properties such as pH, texture, organic carbon and nitrogen can be measured easily compared to the bacterial community structure and so can be carried out for all samples. After reducing the number of samples that have similar properties, the bacterial and/or polyketide synthase communities can be measured by molecular methods.

There are now a broad range of molecular methods that can be used to characterize bacterial communities. In applying these, it is important to use the same DNA extraction and analysis methods so that results from sample to sample are fully comparable. As sequencing is a rapidly developing technology, methods that were once widely used (e.g., 454 pyrosequencing) are now no longer offered [262]. Next generation sequencing can provide a much more detailed view of the bacterial community than earlier methods, through sequencing of tens of thousands of amplicons per sample, or shotgun cloning and sequencing of the metagenome. However these methods often mean that all samples have to be pooled before they are sent for sequencing to keep costs low, which can slow the decision cycle time in sampling/characterizing/resampling interesting habitats.

**Figure 24:**
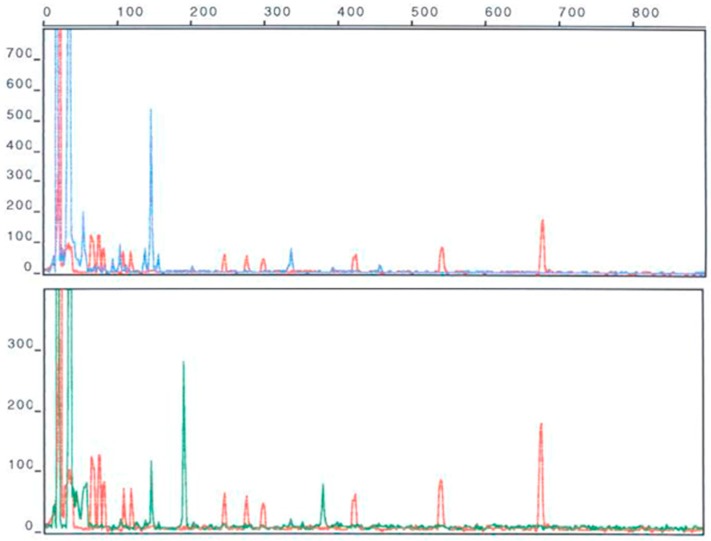
An example of actinobacterial 16S fingerprinting. Forward and reverse Terminal Restriction Fragment Length Polymorphism (T-RFLP) from a Sanger sequencer for a Russian taiga forest soil (Rusforest-Yenisei). PCR products of 16S actinobacterial specific primers were labelled with the dyes hexachloro-6-carboxyfluorescein (blue, forward) and carboxyfluorescein (green, reverse). Red peaks are the ROX 1000 size standards. Size is shown on the X axis in bp, fluorescence on the Y axis. The size range 81–677 bp was used for clustering analysis of forward T-RFLP patterns from a range of samples (shown in Figure 4).

Community fingerprinting may not be able to provide the same level of detail as next generation sequencing, but can quickly pick bacterial communities that are outliers and worth further sampling or more detailed characterization through next generation sequencing. In our experience restriction enzyme fingerprintints such as T-RFLP are difficult to interpret when eubacterial 16S primers are used as many cut sites are the same for different phylogenetic groups, so fingerprints are similar. Using primers that amplify the verrucomicrobia and actinobacteria [263] or the actinobacteria alone [264] (Figure 24) gave community fingerprints that differed from each other [46]. T-RFLP has been successfully used on PKSII sequences from soil [93]. The microbial communities of a subset of these samples can be determined to pick which samples will be used for growing isolates or making metagenomic libraries.

### 4.3. Non Actinomycetal Polyketide Producers

Characterizing environmental DNA with polyketide specific rather than 16S specific primers will give a better view and environments potential for polyketide discovery. 16S specific primers can show how diverse Actinobacteria are and if they are a large fraction of the soil bacterial community. Evidence from type I polyketide specific primers suggests that most PKSI producers in many environments are not actinobacterial.

Whatever their relative importance in the environment, there is a strong argument for focusing on polyketides that appears to be from non-actinomycetal bacteria. Since the mid-1940s pharmaceutical companies have isolated millions of strains of Actinomycetes and tested their secondary metabolites for activity to discover new natural products [36]. Only a very small fraction of these strains has had their genomes or polyketide producing gene clusters sequenced. Thus even novel polyketide sequences from the environment which appears to be actinobacterial may be from an already known polyketide. Non-actinomycetal polyketides on the other hand have not been heavily screened for polyketide production. Since sequencing bacterial genomes has become cheaper, genome mining has found many polyketide pathways in non Actinobacteria. Non actinobacterial genomes may be a better source of genuinely novel polyketides.
